# The Effect of the Move More Pack on the Physical Activity of Cancer Survivors: Protocol for a Randomized Waiting List Control Trial with Process Evaluation

**DOI:** 10.2196/resprot.7755

**Published:** 2017-11-09

**Authors:** Justin Webb, Chris Fife-Schaw, Jane Ogden, Jo Foster

**Affiliations:** ^1^ Centre for Primary Health and Social Care School of Social Professions London Metropolitan University London United Kingdom; ^2^ School of Psychology Faculty of Health and Medical Sciences University of Surrey Surrey United Kingdom; ^3^ Support and Wellbeing Team Macmillan Cancer Support London United Kingdom

**Keywords:** cancer, physical activity, behavior change, health promotion

## Abstract

**Background:**

Physical activity can improve many common side effects of cancer treatment as well as improve physical function and quality of life (QOL). In addition, physical activity can improve survival rate and reduce cancer recurrence. Despite these benefits, only 23% of cancer survivors in England are active to recommended levels. Cancer survivors are interested in lifestyle behavior change. Home-based interventions offer a promising means for changing physical activity behavior. Prediagnosis levels of physical activity and self-efficacy have been reported to be predictors of physical activity behavior change. The Move More Pack, which has undergone revision, is a printed resource with supporting Internet-based tools that aims to increase the physical activity of cancer survivors in the United Kingdom. The revised Move More Pack is underpinned by the theory of planned behavior and the social cognitive theory.

**Objective:**

The aim of this proposed study was to investigate the effect of the revised Move More Pack, supported by Internet-based tools, on physical activity, self-efficacy, and health-related QOL (HRQOL) of cancer survivors in the United Kingdom.

**Methods:**

This study is a two-arm waiting list randomized control trial with embedded process evaluation. A sample of 99 participants per arm will be recruited by invitation through an email database of cancer survivors held by UK charity Macmillan Cancer Support and an advert placed on the Macmillan Cancer Support Facebook page. Each participant is randomized to receive brief physical activity information and the UK guidelines for physical activity, or brief physical activity information and the revised Move More Pack with supporting Internet-based tools. The intervention and control arm will be followed up at 12 weeks to identify changes in self-reported physical activity, self-efficacy, and HRQOL based on Web-based questionnaires. The control arm will receive the revised Move More Pack at 12 weeks with follow-up at 24 weeks. The intervention arm is followed up at 24 weeks to determine maintenance of reported changes. Subgroup analyses will be completed based on participants’ prediagnosis level of physical activity and baseline self-efficacy as possible predictors of positive changes. Use of each component of the revised Move More Pack will be assessed using a 4-point Likert scale. Semistructured phone interviews will evaluate the use and perceived usefulness of the revised Move More Pack.

**Results:**

Participant recruitment started in March 2017. Projected completion of this study is October 2018.

**Conclusions:**

This study’s findings will identify if the proposed low-cost broad reach intervention improves physical activity, self-efficacy, and the HRQOL of cancer survivors. The process evaluation is designed to contextualize the use and perceived usefulness of the revised Move More Pack, help augment its efficient distribution, and identify potential improvements to its design.

## Introduction

### Physical Activity in Cancer Survivors

Two and a half million people are living with or beyond cancer in the United Kingdom [1]. In the last 5 years, this number has grown by almost half a million [1]. The number of cancer survivors, that is, someone living with or after any form of cancer diagnosis [2], is expected to rise to 4 million by 2030 [1].

Developing cancer depends on factors such as age, genetics, and lifestyle behaviors, with a suggested 40% of all cancer diagnosed in the United Kingdom linked to tobacco, alcohol, unhealthy diet, being overweight, and inactivity [3]. Leading a physically active lifestyle reduces people's risk of developing several cancers [4].

Being physically active has multiple benefits for cancer survivors. Physical activity can improve many common side effects of cancer treatments, such as fatigue, psychological distress, and adverse impact on body composition, as well as improving physical function and quality of life (QOL) [5-7]. In addition, increased physical activity is associated with improved survival and reduced disease recurrence [8]. The evidence supports the unequivocal role of physical activity in self-management [5]. Physically active cancer survivors report a sense of regaining control of their lives [9,10] and some normalcy [9-11] following a cancer diagnosis.

Engaging in physical activity is not only recommended but also safe both during and after cancer treatments [7]. The American College of Sports Medicine [7] advises that cancer survivors avoid inactivity and return to typical daily activities as soon as possible after surgery and during and after cancer treatments, working toward the standard age-appropriate physical activity guidelines [7,12]. Despite these benefits, only 23% of cancer survivors in England are active at recommended levels, and 31% are completely inactive [13].

Wang and colleagues [14] report that cancer survivors in Scotland are less likely to smoke, more likely to eat a healthy diet, and more liable to drink alcohol responsibly, although the odds ratios for these conclusions are not compelling. Wang and colleagues [14] also report that cancer survivors in Scotland are less likely to take part in at least 2 hours of physical activity than those who have not had a cancer diagnosis. A cancer diagnosis may offer a teachable moment in which people may be more receptive to changing their lifestyle behaviors [15-18]. McBride and colleagues [18] suggest that low-level interventions may facilitate such an opportunity.

### Self-Efficacy and Self-Identity

Self-efficacy is central in overcoming the barriers faced by cancer survivors in becoming physically active [19]. Self-efficacy is defined in this context as the confidence of a cancer survivor that he or she has the ability and capacity to be more physically active. Self-efficacy has been reported to be a predictor of intentions to change physical activity in cancer survivors [9,20], consistent with the extant general literature on health behavior change [21]. In addition, identifying as a physically active individual has been reported to be an indicator of physical activity engagement, with those cancer survivors who are physically activity before their diagnoses being more likely to be so afterwards [9]. However, physical activity tends to decrease following diagnosis [22] and is unlikely to reverse without intervention. These predictors of physical activity are reported to be consistent across cancer survivors regardless of age, stage, type of cancer, comorbidity, or treatment received [9].

### Remote Support to Facilitate Physical Activity Behavior Change

Cancer survivors report a high level of interest in lifestyle interventions [20,23-25]; however, access to face-to-face programs is not always possible because of transportation issues and geographic and access considerations [24]. There is a demand for written health information to support behavior change [24,26-28]. Advantages include message consistency, ease of delivery, self-paced learning, and the permanence of information with low production costs [29].

Home-based interventions using printed materials offer a cost-effective, potentially promising means of intervening regardless of location [23,24]. At a time when spending on public health and health care in the United Kingdom continues to be constrained, with demand for services increasing, the need for home-based interventions is growing. Randomized control trials (RCTs) have been, and continue to be, conducted on the efficacy of such interventions [23,30]. However, none has included a process evaluation to contextualize the use of such interventions in a real-world setting removed from the health care environment.

### The Move More Pack and its Revision

The UK charity, Macmillan Cancer Support, developed a printed resource in 2011 called the Move More Pack that aimed to effect change in physical activity in cancer survivors. The Move More Pack consisted of a physical activity and cancer booklet and a series of written assignments to support behavior change. No additional assistance or follow-up was provided. The effectiveness of the Move More Pack in effecting change in physical activity in cancer survivors has not yet been investigated.

The principal investigator led the redevelopment of the Move More Pack in 2016 to become a printed resource supported by a series of Internet-based tools. Following discussions with cancer survivors [9,31], a systematic search and critical appraisal of the literature, a review of the original Move More Pack with respect to its underlying theoretical constructs, and an inventory of the behavior change techniques (BCTs) it advocates, recommendations were made.

The theory of planned behavior (TPB) [15,32] and the social cognitive theory (SCT) [33-35] were identified as appropriate theories upon which to base the redevelopment of the Move More Pack. The original Move More Pack was assessed using the constructs of the TPB and the SCT. The BCT taxonomy version 1 (BCTTv1) [36] identified the active ingredients that aimed to effect change and those that might be missing from the original design. Following an iterative process, a group of six subject experts including the principal investigator, in partnership with Macmillan Cancer Support’s information development team, refined the structure, content, and BCTs included within the revised Move More Pack. Macmillan Cancer Support’s information development team wrote the final copy of the revised Move More Pack. Dr Tim Iverson, Macmillan Cancer Support’s chief medical editor, approved the final version. The revised Move More Pack received the National Health Service England (NHS England) Information Standard [37]. A PDF of the revised Move More Pack is included as Multimedia Appendix 1.

The revised Move More Pack retained the physical activity and cancer booklet (Multimedia Appendix 2). A pull-out wall chart is included in the revised Move More Pack for users to track their progress, record achievements, and serve as a visual prompt to be more active (Multimedia Appendix 3). Furthermore, five activity leaflets (Multimedia Appendix 4) are included on popular activities of gardening, walking, and recreational swimming; the sports of badminton, bowls, cycling, golf, and walking football; and finally, on how to be generally active in daily life [38]. A digital versatile disc that focuses on exercise specifically for cancer survivors is included and is also available to view on the Web [39].

Tailored multi-component interventions are likely to be most effective in effecting lifestyle behavior change in cancer survivors [35,40,41]. Users of the revised Move More Pack can sign up to receive e-newsletters, with messages tailored to their reported prediagnosis levels of physical activity, and influenced by the stages of physical activity behavior change advanced by Marcus and Forsyth [42]. Prediagnosis levels of physical activity are collected using the Godin Leisure Time Exercise Questionnaire (GLTEQ) [43]. Case studies included within the e-newsletters are tailored to the age and gender of the user of the revised Move More Pack. A welcome email is sent to users of the revised Move More Pack, followed by e-newsletters sent during months 1, 2, 3, 6, 9, and 12. Details are outlined in Table 1, with an example e-newsletter included as Multimedia Appendix 5.

An online social community aims to link users of the revised Move More Pack, enabling social learning and enhancing social norms [44]. An online *ask the physio* group is available and allows users of the revised Move More Pack to post questions on an open forum to a registered cancer specialist physiotherapist [45]. Details of how to find local physical activity opportunities are also included on the Web [46].

The use of a pedometer combined with a printed resource has been reported to be effective in increasing physical activity in breast cancer survivors [32]. Consequently, details are provided on how to download a straightforward and easy to use digital pedometer app, as well as an app to reduce sitting time [47]. Finally, a series of video case studies of cancer survivors who have become more active are included [48]. A Web page dedicated to users of the revised Move More Pack links to these Internet-based tools (Multimedia Appendix 6) [49].

The revised Move More Pack and Internet-based tools have been developed based on the best available evidence and following guidance on the development of complex interventions from the United Kingdom based Medical Research Council (MRC) [50]. The revised Move More Pack does not prescribe physical activity; rather, it aims to empower cancer survivors to increase control over their physical activity behavior.

### Aims

This study aims to investigate the effect of the revised Move More Pack over 24 weeks. It is hypothesized that use of the revised Move More Pack increases physical activity in cancer survivors, and the proportion of cancer survivors who are classified as active over 12 weeks increases with its use, with changes being maintained at 24 weeks. The primary aim is to test the effectiveness of the revised Move More Pack in reclassifying cancer survivors categorized as inactive or moderately active at baseline, to being more active over 12 weeks, with increases maintained at 24 weeks.

The secondary aims include:

- Test the effect of the revised Move More Pack on the self-efficacy and health-related QOL (HRQOL) of cancer survivors.
- Analyze subgroups to elucidate for whom the revised Move More Pack has a positive effect on physical activity, self-efficacy, and HRQOL in the context of prediagnosis levels of physical activity and baseline self-efficacy.
- Conduct a process evaluation to contextualize the use and perceived usefulness of the revised Move More Pack.

**Table 1:** The theme and behavior change techniques used in the e-newsletters sent to users of the revised Move More Pack.

Newsletter		Stage of change^a^	BCTs^b^ used^c^
**For those active before diagnosis**^d^			
	Month 1	Doing some physical activity	Information about others’ approval; Information about health consequences; Information about emotional consequences; Graded tasks; Social comparison; Goal setting (behavior)
	Month 2	Doing some physical activity	Information about others’ approval; Information about health consequences; Information about emotional consequences; Framing or reframing; Graded tasks
	Month 3	Doing enough physical activity	Information about others’ approval; Social support (unspecified); Self-reward; Action planning
	Month 6	Making physical activity a habit	Self-monitoring; Action planning; Habit reversal; Habit formation
	Month 9	Making physical activity a habit	Self-monitoring; Action planning; Habit reversal; Habit formation; Social support (unspecified)
	Month 12	Making physical activity a habit	Self-monitoring; Action planning; Habit reversal; Habit formation; Social support (unspecified); Self-reward
**For those inactive before diagnosis**^d^			
	Month 1	Inactive and thinking about becoming physically active	Information about others’ approval; Information about health consequences; Information about emotional consequences; Graded tasks; Social comparison
	Month 2	Doing some activity	Information about others’ approval; Information about health consequences; Information about emotional consequences; Framing or reframing; Graded tasks
	Month 3	Doing some activity	Information about others’ approval; Goal setting (behavior); Self-reward; Action planning; Commitment
	Month 6	Doing enough physical activity	Self-monitoring; Action planning
	Month 9	Making physical activity a habit	Self-monitoring; Action planning; Habit formation; Social support (unspecified)
	Month 12	Making physical activity a habit	Self-monitoring; Action planning; Habit reversal; Habit formation; Social support (unspecified); Self-reward

^a^On the basis of the stage of change constructs offer by Marcus and Forsyth [42].

^b^BCT: behavior change technique.

^c^BCT selected from the BCT Taxonomy version 1 [36].

^d^Prediagnosis levels of physical activity assessed using question two of the Godin Leisure Exercise Time Questionnaire.

## Methods

Identifying the underlying theoretical constructs of a health-related program is key when designing its evaluation [51]. A breakdown of the components of the revised Move More Pack based on the constructs of the SCT and the TPB, along with the BCTs used in available as Multimedia Appendix 7.

### Design

**Figure 1:**
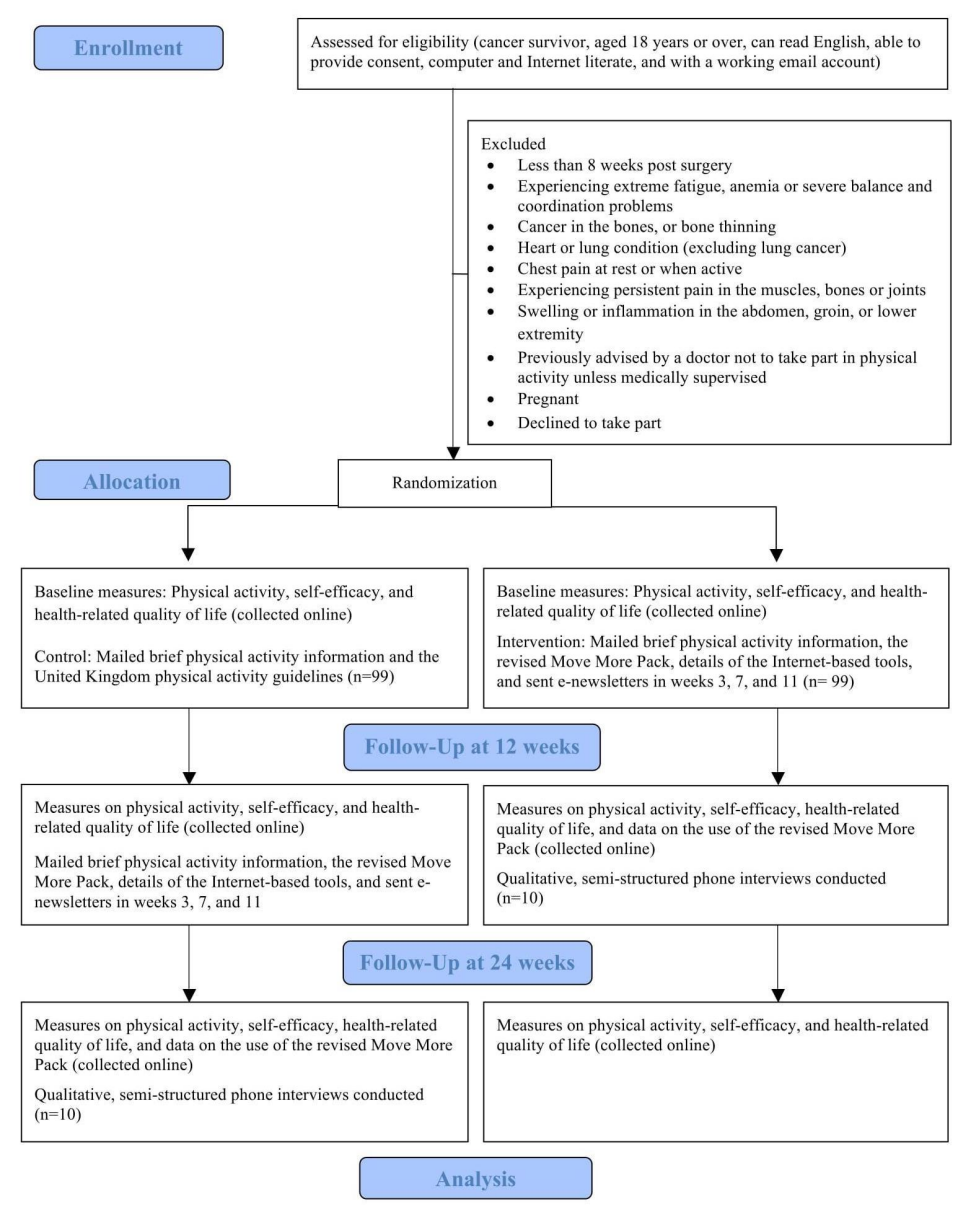
Participant flow through this waiting list randomized control trial with embedded process evaluation.

This study is a two-arm waiting list RCT (ISRCTN 66418871) with embedded process evaluation, designed following guidance from the MRC for evaluating complex interventions [52]. Figure 1 shows the progress through the phases of this study. The control arm participants receive brief physical activity information and details of the UK guidelines for physical activity. Intervention arm participants receive brief physical activity information, the revised Move More Pack, directions to the Internet-based tools, as well as the e-newsletters as outlined in Table 1, up to and including the newsletter sent in month 3.

### Sample size

NHS England [13] report that 23% of cancer survivors are active to the recommended levels for aerobic activity, 31% are inactive, and 46% are physically active but not to the recommended levels. NHS England [13] also reported that 18% of cancer survivors are interested in becoming more active. The sample size for this study has been calculated based on the assumption that the revised Move More Pack will increase the proportion of the sample achieving the aerobic physical activity guidelines by 18%. A sample of 82 participants will be required per arm for a one-tailed test, power of 80% with alpha set at 5%. A total of 99 participants will be recruited per arm to allow for a 20% dropout.

### Recruitment

Participants will be recruited by email invitation through the Macmillan Cancer Support database of cancer survivors. An advert will also be placed on the Macmillan Cancer Support Facebook page [53]. Those that express an interest will be sent further participant information by email, with consent provided digitally by check box, following the British Psychological Society ethics guidance for Internet-mediated research [54]. Participants will be informed that the study aims to investigate the impact of health promotion information on lifestyle behaviors, with no specific reference made to physical activity. Participants will be notified that they will be randomized to receive guidelines for a lifestyle behavior or a health promotion pack with Internet-based tools relating to a lifestyle behavior. Recruited participants will be randomized based on simple randomization. Recruitment will continue until 99 participants are randomized to either the control or intervention arm.

### Inclusion Criteria

This study will include cancer survivors regardless of cancer stage, cancer type, or comorbidity. Participants will be aged 18 years or over, who can read English, can provide consent, are computer and Internet literate, and have a working email account.

### Exclusion Criteria

There are greater risks from being inactive than taking part in physical activity. The revised Move More Pack does not prescribe exercise in any way, and the relevant safety information is sent in the post to participants at the start of the study. The safety information is taken from the Macmillan Cancer Support Web pages [55] and has received the NHS England Information Standard [37]. However, those participants considered at high risk of injury are excluded from this study. On the basis of guidance on exercise and cancer survivorship from the American College of Sports Medicine [7,56], reviewed and approved by subject experts from Macmillan Cancer Support’s physical activity team, the following screening questions will be asked of participants, with an answer of yes to any question resulting in exclusion from the study:

- Are you less than 8 weeks postsurgery?
- Are you experiencing extreme fatigue, anemia, or severe balance and coordination problems?
- Do you have cancer in your bones or bone thinning?
- Do you have a heart or lung condition (excluding lung cancer)?
- Do you feel pain in your chest at rest, during your daily activities, or when becoming active?
- Do you have persistent pain in your muscles, bones, or joints?
- Do you have swelling or inflammation in the abdomen, groin, or lower extremity?
- Has your doctor ever said that you should only do medically supervised physical activity?
- Are you pregnant?

Excluded participants will be informed that they will need medical approval before becoming more physically active and, therefore, are not eligible for this study. They will be thanked for their time and given the details of how to order the free resources offered to participants in this study, for use after receiving permission from their general practitioners or cancer care teams, should they decide to become more active.

### Procedures and Assessment Tools

#### Effectiveness

Physical activity will be assessed using the GLTEQ, a reliable and validated tool [43] used previously with cancer survivors [57]. The cancer specific 7-item Functional Assessment of Cancer Therapy Questionnaire (FACT-G7), also a reliable and validated tool, will be used to assess HRQOL [58].

The GLTEQ and the FACT-G7 will be administered electronically at baseline in the intervention and control arms of the study. Participants will be asked to complete the GLTEQ twice:

1. To consider their levels of activity in a standard week before their cancer diagnosis, to allow for the tailoring of the e-newsletters, and to provide a context for the use of the revised Move More Pack.
2. To consider their levels of activity in a standard week after diagnosis, as a baseline assessment of physical activity.

Self-efficacy will be assessed using the following single-item assessment tool: “On a scale of 1 to 10 (1=not at all confident and 10=very confident), how confident are you that you will be physically active in situations such as the following: feeling tired, bad mood, not having the time, on vacation, bad weather?” A similar measure has been used previously with cancer survivors [59]. A single-item assessment tool is selected for its practical application to a real-world setting, and furthermore, single-item assessment tools can perform just as well as multi-item assessment tools [60].

Additional participant information will be collected on date of birth, gender, primary cancer site (type), time since diagnosis, treatment received, time since completion of treatment, response to treatment, and ethnic group. The structure of these questions is that used by NHS England [13]. These questionnaires and participant data will be collected using software from Qualtrics, USA.

#### 12-Week Follow-Up

The effectiveness of the revised Move More Pack at effecting change in physical activity, self-efficacy, and HRQOL in the intervention arm will be evaluated after 12 weeks using the assessment tools used at baseline. The control arm will also be assessed at this 12-week time point. Participants will not have access to their previous scores.

At the 12-week time point, participants in the control arm will be mailed the revised Move More Pack, directed to the Internet-based tools, and will receive the e-newsletters as outlined. The control arm will be followed up a further 12 weeks later, at 24 weeks, to evaluate change in physical activity, self-efficacy, and HRQOL. Participants in the intervention arm will continue to have access to the Internet-based tools, although they will no longer receive e-newsletters after the 12-week time point.

#### Maintenance

The maintenance of reported changes in physical activity, self-efficacy, and HRQOL for participants in the intervention arm will be evaluated after 24 weeks with the same assessment tools. Participants will not have access to their previous scores.

Participants will be informed that they can withdraw from the study at any time by contacting the principal investigator. In such cases, the reason for withdrawal from the study will be ascertained and recorded. Nonresponders to the questionnaire will be followed up by email to record their reasons for dropping out of the study.

#### Process Evaluation

Use of each component of the revised Move More Pack will be assessed using a 4-point Likert scale of often, sometimes, rarely, and never. The 4-point Likert scale is included as part of the questionnaire administered to the intervention arm at 12 weeks and the control arm at 24 weeks. Participants will also have the opportunity to add comments about their use and their perceived usefulness of the revised Move More Pack. At the 12-week time point, participants from the intervention arm will be stratified into two groups, those inactive before diagnosis and those moderately active or active before diagnosis. Five participants from each group will be randomly selected and interviewed by phone to gain a deeper understanding of their interaction with, and views of, the revised Move More Pack. This interview process is repeated in the control arm at 24 weeks.

Interviews will follow a semistructured format and will be conducted by the principal investigator. This format has been selected to ensure that data are collected on central topic areas of use and perceived usefulness of the components of revised Move More Pack while not restricting the flow of the conversation. In addition, the interviews will aim to gather data to situate the experience of using the revised Move More Pack within a broad social context. The interview topic guide is included as Multimedia Appendix 8.

### Data Analysis

The quantitative data will be analyzed using intention-to-treat analysis. The GLTEQ provides a physical activity score, and these scores can be used to categorize participants into inactive, moderately active, and active groups. The two-proportion *z* test is used to investigate differences between the proportions of participants in the control arm and the intervention arm classified as active.

Secondary analysis of the paired before and after ordinal data within each intervention arm will be analyzed with the Wilcoxon’s signed-rank test; across arms, comparisons will be analyzed with the Mann-Whitney *U* test. The mean GLTEQ scores will be analyzed with the independent *t* test and the paired *t* test. The predictors of self-efficacy and prediagnosis physical activity on post-intervention physical activity levels will be assessed by multiple regression analysis. Additional quantitative data will include descriptive statistics such as means, standard deviations, medians, and percentages.

The interviews will be recorded and transcribed verbatim. The interview transcripts and the qualitative comments made by the participants as part of the questionnaires administered at the 12-week and 24-week time points will be thematically analyzed [61] by two of the study investigators, ensuring that identified themes are grounded in the original data. A third investigator will be used where there are differences in opinion. The process of analysis will follow five stages:

1. Familiarization with the data involving reading and rereading the interview transcripts and qualitative comments.
2. Initial coding.
3. Theme identification.
4. Theme review and development of higher level categories.
5. Identification of relationships and patterns.

The investigators will move back and forth through these steps until they concur and are satisfied with the themes, categories, relationships, and patterns identified.

### Cost Consequence Analysis

The economics of the revised Move More Pack will take the form of a cost consequence analysis, with costs assessed against a range of outcomes. Although this will not draw definitive conclusions regarding cost effectiveness, it will identify the costs of achieving the reported outcomes. The development costs and other costs needed to make the revised Move More Pack usable will not be included.

### Data Management

Web-based questionnaires will be completed using software from Qualtrics, USA. The questionnaire software from Qualtrics, USA, treats data as highly confidential [62] and offers the highest levels of data security [63]. Ownership, control, and management of data remain with the University of Surrey.

Information gathered will be secured on password-locked computers and the servers at the University of Surrey. Hard files will be stored in locked cabinets within the university. Project data, for example, consent forms, will be retained for at least 6 years and research data for at least 10 years as stipulated by the policies of the University of Surrey [64]. Personal data will be secured and processed in the strictest confidence according to the Data Protection Act [65].

Data for analysis and reporting is anonymized. Identifiable data are accessible only by the principal investigator, members of the research team, and authorized personnel from the University of Surrey, and regulatory authorities for monitoring purposes.

### Ethical Considerations

The information included in the revised Move More Pack is certified by the NHS England Information Standard [37]. The NHS England Information Standard ensures that publically available information has undergone rigorous assessment, is evidence-based, of high quality, clear, accurate, and appropriate for its intended audience. The revised Move More Pack does not prescribe exercise. The relevant safety information is sent to participants in the first postal communication. Criteria for cessation of physical activity is provided, for example, sudden onset of dizziness, chest pains, a racing heartbeat, breathing problems, nausea, unusual back or bone pain, muscle weakness or a persistent headache, advising participants to contact their doctors for these or other symptoms. Appropriate screening is in place within the study procedures to identify participants needing medical permission before increasing their physical activity, with these participants being excluded from this study. A log of participant issues will be maintained throughout the study, and participants will be offered a phone debriefing session at the end of the study.

Participants in the control arm will not be restricted with regard to being physically active. The participants in the control arm will be asked as part of the questionnaire instructed at the 12-week follow-up time point if they have used the revised Move More Pack within the previous 12 weeks, with their data omitted from the study if they have. This study received ethical approval from the University of Surrey Research Ethics Committee on March 15, 2017, reference UEC/2017/023/FHMS.

## Results

Recruitment for this study began in March 2017. This study has a projected completion date of October 31, 2018.

## Discussion

With improvements in treatment, people are now living longer with cancer, and the condition is now in many cases classified as chronic [1]. Cancer survivors, like others with long-term conditions, are heavy users of the NHS. Seventy percent of the NHS’s spending is on the 15 million people living with long-term conditions [66]. However, less than 1% of their time is spent in contact with health care professionals [67]. Becoming more physically active has been shown to have many benefits for cancer survivors and has a key role in supporting the self-management of the consequences of cancer and its treatments.

There is a lack of reporting within the literature on how effective physical activity interventions for cancer survivors are developed and designed and their impact on physical activity, self-efficacy, and HRQOL [68]. To the knowledge of the research team, this is the first intervention to combine a printed physical activity behavior change pack with Internet-based tools, including online access to a cancer specialist physiotherapist, to increase physical activity, self-efficacy, and HRQOL in cancer survivors.

Printed materials supported by Internet-based tools are likely to provide a low-cost approach to physical activity behavior change. The process evaluation will contextualize the use and perceived usefulness of the revised Move More Pack, which will augment efficient distribution and identify needed improvements to its design. The revised Move More Pack may offer some promise as a first line intervention to improve the lifestyles of cancer survivors, particularly in relation to physical activity and exercise. If a marked effect size can be demonstrated, the revised Move More Pack could well provide considerable cost-saving to the overstretched NHS funding in the United Kingdom.

The major limitation of this study is the use of self-reported measures to assess and evaluate participants’ physical activity, self-efficacy, and HRQOL. The measures selected are validated and reliable. The GLTEQ has been used in previous research with cancer survivors [57], and the FACT-G7 is specifically designed for cancer survivors [58]. The self-reported measures have been selected for their ease of implementation in a real-world setting. Whereas the use of an objective measure of physical activity may be preferable, this would introduce an additional behavior change technique. Furthermore, as the revised Move More Pack aims to enable cancer survivors to monitor their physical activity by directing them to use a pedometer, introduction of an objective measure may influence the effectiveness of this component of the revised Move More Pack.

Some studies of cancer survivors have reported high levels of dropout [69]. Therefore, an additional 20% will be recruited to this study. Furthermore, a combination of strategies will be employed to encourage participants to complete the relevant questionnaires at the data collection points, including email and text reminders.

It is possible that the exclusion criteria for this study may result in a selection bias; however, the safety of the participants will not be compromised. In addition, as participants are recruited through the channels of Macmillan Cancer Support, they may not be representative of the population of cancer survivors in the United Kingdom. The profile of the included participants will be reported and any selection bias identified.

## Appendix

Multimedia Appendix 1 The revised Move More Pack.

Multimedia Appendix 2 The Physical Activity and Cancer booklet.

Multimedia Appendix 3 Pull-out wall chart.

Multimedia Appendix 4 Physical activity leaflets.

Multimedia Appendix 5 Example e-newsletter.

Multimedia Appendix 6 Web page dedicated to users of the revised Move More Pack.

Multimedia Appendix 7 A breakdown of the component of the revised Move More Pack.

Multimedia Appendix 8 Interview topic guide.

## Abbreviations

BCT: behavior change technique
BCTTv1: behavior change technique taxonomy version 1
DVD: digital versatile disc
FACT-G7: 7-item Functional Assessment of Cancer Therapy Questionnaire
GLTEQ: Godin Leisure Time Exercise Questionnaire
MRC: Medical Research Council
NHS England: National Health Service England
RCT: randomized controlled trial
SCT: social cognitive theory
TPB: theory of planned behavior
QOL: quality of life
HRQOL: health-related quality of life
