# Supplementary material for: The Effect of the Move More Pack on the Physical Activity of Cancer Survivors: Protocol for a Randomized Waiting List Control Trial with Process Evaluation
Source: JMIR Res Protoc. 2017 Nov 9;6(11):e220. doi: 10.2196/resprot.7755 (PMC5701086; doi:10.2196/resprot.7755)
Supplement: Multimedia Appendix 1 [file resprot_v6i11e220_app1.pdf]

WE ARE MACMILLAN. CANCER SUPPORT

A practical guide to living with and after cancer

MOVE MORE: YOUR GUIDE TO BECOMING MORE ACTIVE

About this guide

If you've had a cancer diagnosis and want to make a positive change to your life, this guide will help you do just that.

Taking part in physical activity before, during and after cancer treatment can help you take back control. Lots of people say it helps them feel more like their old self. It also has lots of health benefits. It can help prevent and manage some of the effects of treatment, such as fatigue, depression and risks to your heart health.

This is your step-by-step guide to becoming more active.

Here you'll find tips on choosing which activity to do and where to get support. If you haven't been active for a while or are new to being active, this guide will help you set realistic and achievable goals. We've included some tips to help you get started, information on how to set goals and an **activity diary** to help you keep track of how you're doing.

Many people find it helps to get active with a family member or close friend. You could work through this guide with them, to make getting active part of your time together.

Throughout this guide we've included some quotes from people affected by cancer, who have found getting active has helped them. These are from people who have chosen to share their story with us. To share your story, visit **macmillan.org.uk/cancervoices**

Inside the front pocket of this guide

You'll find lots of useful information to help you become more active, including the following:

- **Physical activity and cancer** booklet – this has information about the benefits of being physically active and the types of activity that may be right for you.
- Five short flyers about some popular types of physical activity – **Activity in daily life**, **Walking**, **Swimming**, **Gardening** and **Sport**.
- **Activity chart** – you could use the magnet to put this on your fridge so you can see what you've achieved each day.
- **Move more DVD** – this has gentle activity videos you can do at home.

Support online

We also have lots of useful information on our website at **macmillan.org.uk/beactive** Here you can:

- sign up to our regular newsletters for more advice and tips about becoming active
- find activities in your local area
- ask our experts questions about being active
- join our online community to share your experiences and meet others who have used this guide to become more active.

If you would like to discuss this information, call the Macmillan Support Line free on **0808 808 00 00**, Monday–Friday, 9am–8pm. If you're hard of hearing, you can use textphone **0808 808 0121**, or Text Relay. If you'd prefer to speak to us in another language, interpreters are available.

Contents

Why get active? 5

Getting started 9

Setting goals and staying active 23

‘It’s for your mental health as much as physical health. Having a positive attitude and being physically well helps you combat what has gone on before and after the cancer.’

Clare, diagnosed with breast cancer in 2011

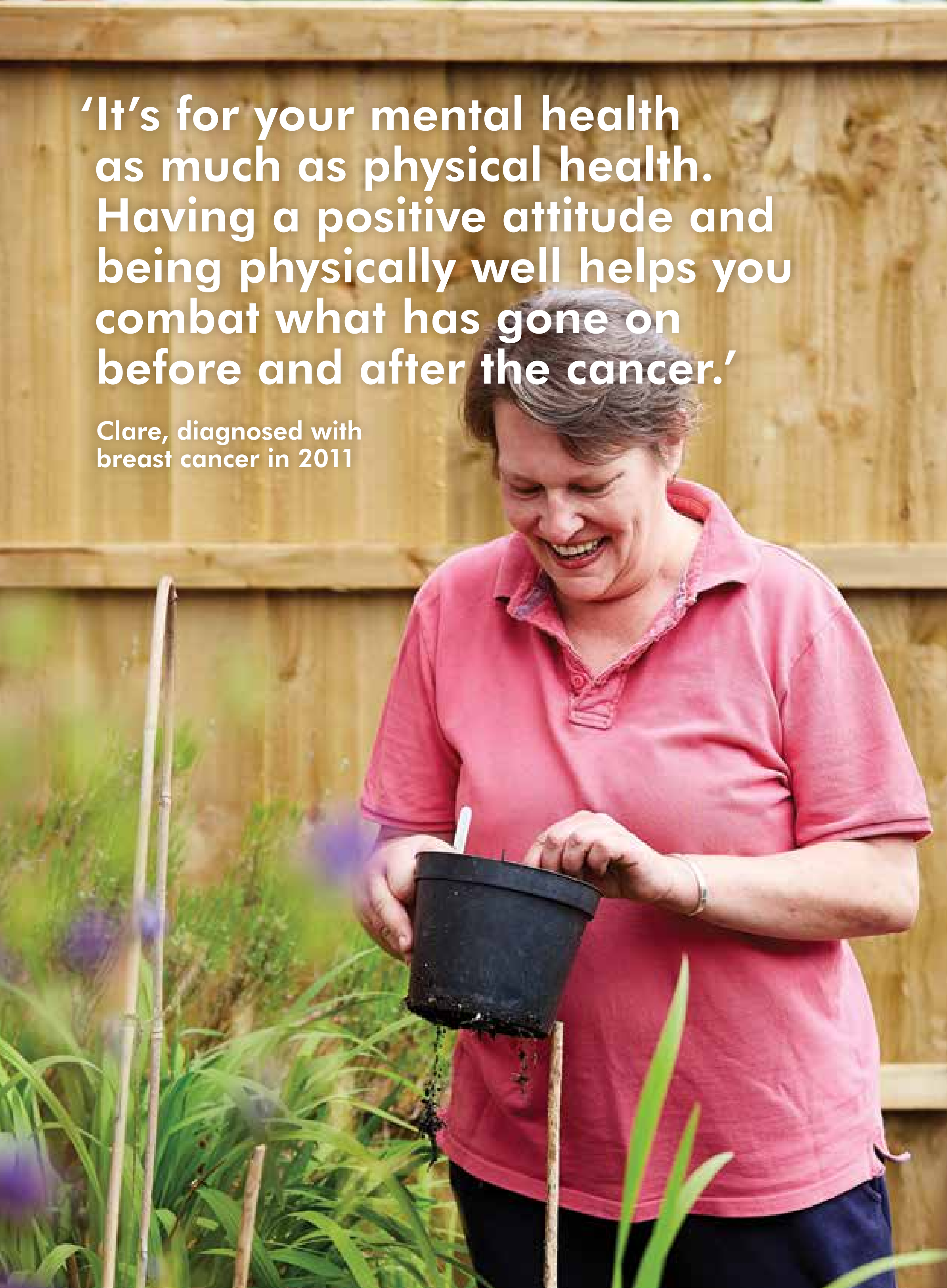

WHY GET ACTIVE?

About physical activity 6

About physical activity

Physical activity is about moving more. This could be everyday activities like walking and housework, as well as more structured exercise like fitness classes at the gym. There are lots of ways you can begin to be more active, and even the smallest increase can improve your quality of life.

You'll find all the details about the benefits of moving more in our **Physical activity and cancer** booklet included in the front of this guide. The booklet also has more information about the types of activity that may be right for you and how much to do.

The types of activity and how much you choose to do will depend on your interests and your current level of activity. It will also depend on your individual situation, for example your cancer type, your treatment and any other long-term conditions you may have.

Remember you can ask your cancer doctor, your specialist nurse or your GP for advice about becoming more active. They may refer you to an exercise specialist or physiotherapist.

We run regular 'ask the experts' sessions on our website where you can ask questions about being active. Visit **macmillan.org.uk/beactive** to get involved. You can also share your experiences and chat to other people affected by cancer on our online community at **macmillan.org.uk/community**

One-to-one activity support services can help you become more active

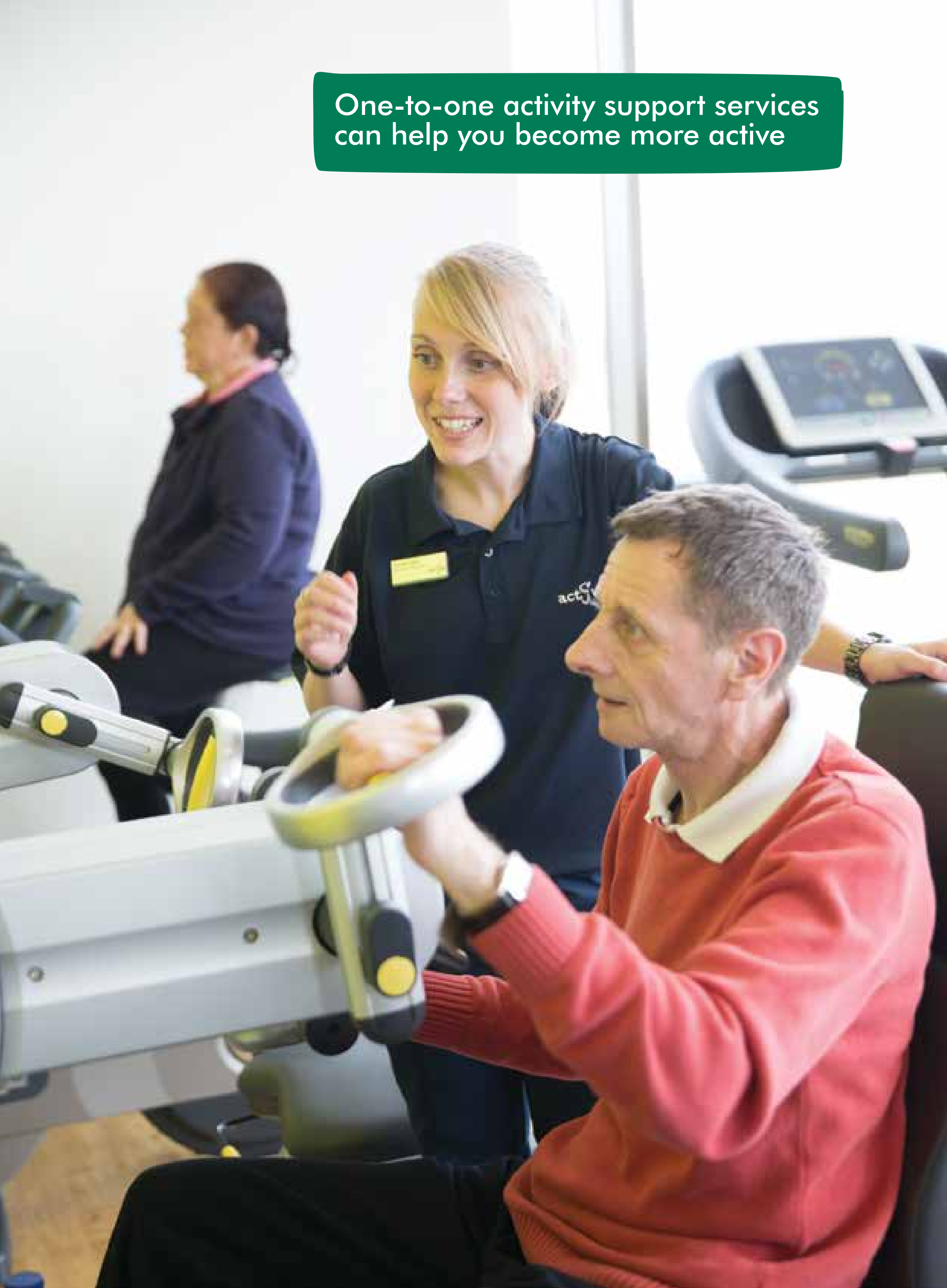

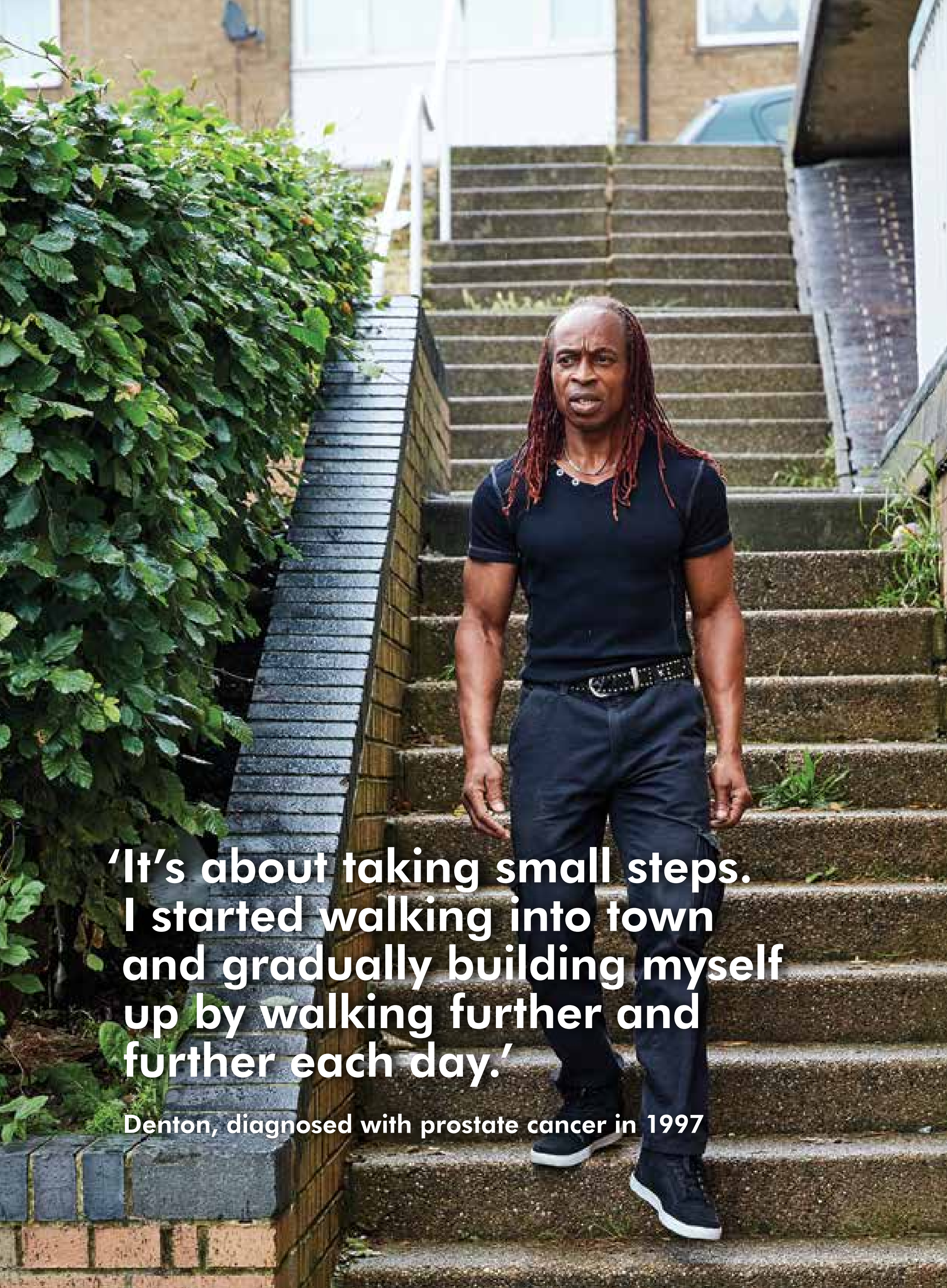

‘It’s about taking small steps. I started walking into town and gradually building myself up by walking further and further each day.’

Denton, diagnosed with prostate cancer in 1997

GETTING STARTED

Questions to help you get started 10
Simple ways to be more active 13
Which activities are right for you? 16
Physical activity in your area 18
Ten top tips 19

Questions to help you get started

When you're making an important lifestyle change, it can be helpful to think about why you're doing it and what you'd like to achieve.

Having someone to be active with can help to motivate you. You might want to talk about these questions with family or friends to help you get started.

What are your top three reasons for becoming more active?

Many people find it helpful to think about their own personal reasons for becoming more active. This could be something as simple as 'I want to improve my mood', or 'I want to do everyday things that I used to take for granted'. You can use the space below to write these down.

1.

2.

3.

Reminding yourself of these reasons when you set your future goal (see pages 24–25) could help keep you motivated.

How might life be different?

Looking forward a few weeks or months, think about how life might be if you became more active. Write down the good things you might feel, or what might be different for you. Then imagine if you didn’t take the opportunity to become more active and what your worries might be.

We understand you might find this difficult or emotional, so you could think about asking someone you trust to help you.

What good things could come out of being more active?	What would life be like if you didn’t become more active?
For example, ‘I would feel more in control’, ‘I would feel more independent.’	For example, ‘I would need to rely on other people to help me get around’, ‘I wouldn’t have much energy’.

Now make a commitment to moving more.

Share your commitment with your family or friends, so you can move more together.

The steps on the following pages will help you meet your commitment and start moving more. Completing the action plan on pages 34–35 will also help bring your thoughts and plans together.

'The first problem I wanted to sort out was the early-morning depression. I now do a lot of walking and when I come in from that the depression's gone for the day.'

Alfie, diagnosed with prostate cancer in 2010

Simple ways to be more active

There are lots of ways you can become more active. It's important to do something you enjoy and that fits in with your life.

This could be walking to the shops, playing with your children or grandchildren in the park, gardening or going dancing with friends. Or it may mean spending less time sitting or lying down, and being more active around the home. Everyone's experience of cancer is different, both during and after treatment. Listen to your body to see which activities feel right for you.

There are lots of ways you can become more active, including:

- doing more around the home, such as housework or gardening
- walking or cycling to work, to the shops, to see friends, or on the school run
- joining a walking group, or using a pedometer to record your steps each day – visit **macmillan.org.uk/beactive** for information on how to order one
- stretching exercises like yoga, tai chi or pilates
- activities that can be done with friends and family, such as swimming or dancing
- doing the activities on our **Move more DVD** – included in the front of this guide
- taking up a sport like badminton, bowls or golf.

You could also ask your GP if you can be referred to a structured exercise programme for people with long-term conditions. This is usually based at a gym.

Macmillan has developed physical activity support services to help you become more active. These services give you the opportunity to discuss your needs with a cancer and physical activity specialist. The specialist will help you set manageable goals and give you ongoing support. They will help you find activities that are right for you. These services are available in a limited number of places around the UK. To find out if you have one in your area, visit **macmillan.org.uk/beactive**

You can read more about the activities that may be right for you on pages 24–34 of our booklet **Physical activity and cancer**. The five flyers included in the front pocket of this guide also give information about some of the most popular types of physical activity, including walking, swimming and cycling.

‘Joining an exercise class has really given me a lift. I was feeling very lonely, but now I’ve got a reason to leave the house and see other people.’

Billy, diagnosed with colon cancer in 2014

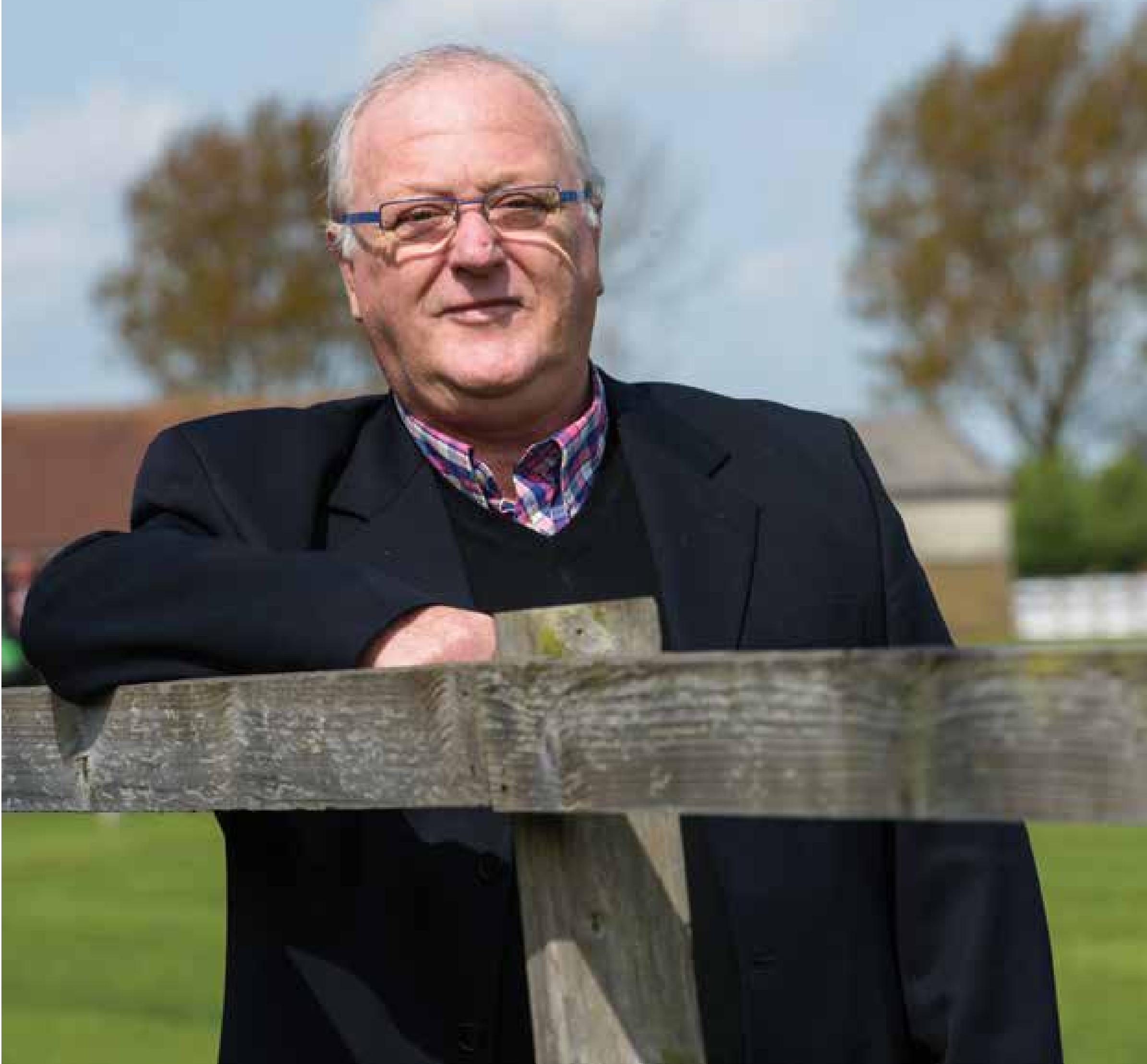

Which activities are right for you?

Becoming active for the first time or returning to activity during or after a condition like cancer isn't always going to be easy. Before becoming more physically active, try to plan which activities you would like to do that fit with your lifestyle and that you enjoy.

If you are going through treatment or feel fatigued (extreme tiredness) at a particular time of day, you may find it useful to plan your activity around this.

'I thought I would never be able to do all the things that I previously enjoyed following cancer, but setting myself targets to be the best that I can be physically has resulted in me getting my life back and more. I cannot run and never will be able to! But I can walk, which is better as I can enjoy what is around me more.'

Lorraine, diagnosed with lung cancer in 2007

Think about what you are most interested in doing and what you would enjoy the most. Make a list here of the different activities you and your family and friends could try.

Physical activity in your area

There are a number of ways to find out which activities are available near you. Visit **macmillan.org.uk/beactive** for more information and useful links.

You may also find it helpful to read the information on pages 42–45 of the **Physical activity and cancer** booklet, in the front of this guide.

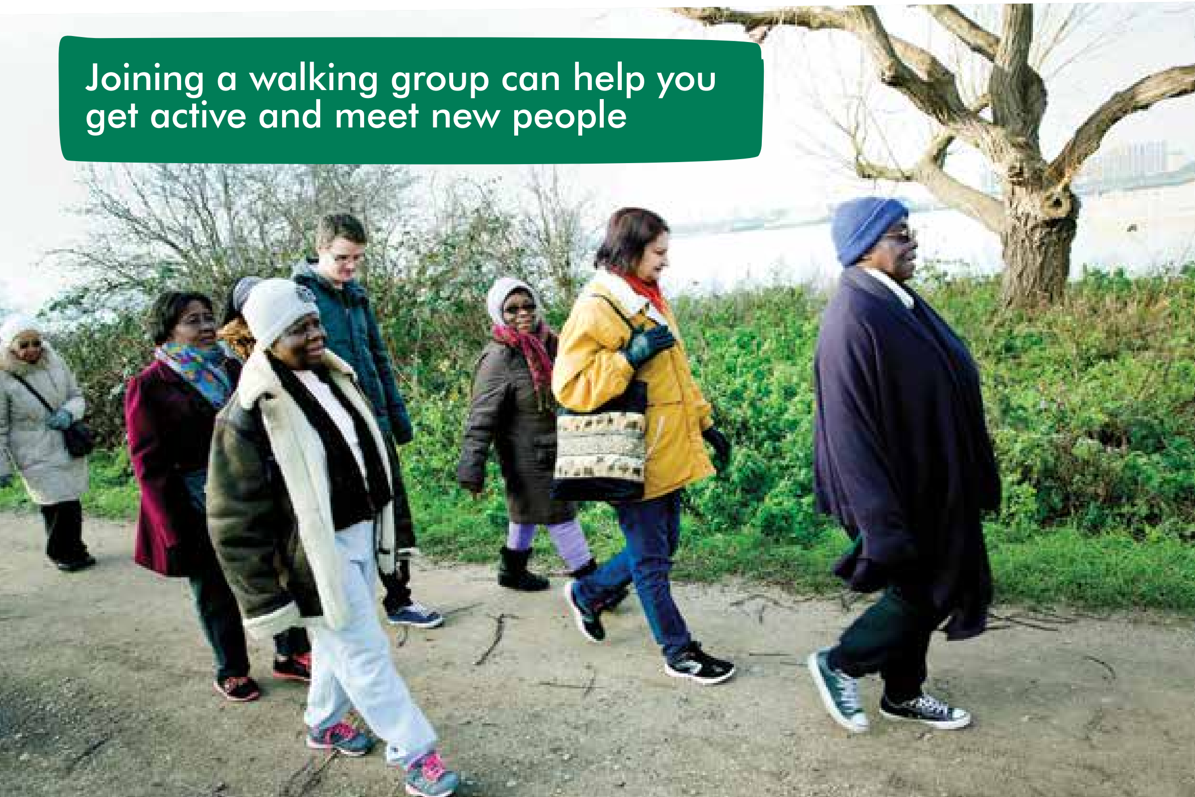

Joining a walking group can help you get active and meet new people

Ten top tips

Getting active may be a big lifestyle change for you, and being active during or after a condition like cancer may not be easy. Here are some top tips on how to get started and keep going:

1. Think about the benefits

Keep in mind what you hope to achieve from becoming more active, and the benefits for your health.

2. Set achievable goals

Whether it's being more active around the house, going for a walk or participating in a class, make sure it's the right goal for you and work towards it at your own pace. Remember, even just preparing to be a more active you is a goal.

3. Don't get disheartened

If you don't achieve a planned goal, think about what went wrong and set a new goal.

4. Build up gradually

Don't try to do too much too soon.

5. Track your progress

Keep a record of how active you've been and how you felt afterwards. You could use the activity diary on pages 39–54 to do this. You might also find it useful to use a pedometer to track how many steps you do each day.

6. Make it social

Try being active with other people, such as family or friends. Or you could join a group or a club. Share your plans with other people who are supportive.

7. Enjoy yourself

Find activities that are right for you and that you enjoy.

8. Try new activities

Some people find that once they feel they can do more, trying something different helps them stay motivated.

9. Make it a habit

Try to incorporate more activity into your daily life. You could sit less, take the stairs, or walk and carry the shopping instead of taking the car.

10. Reward yourself

It's important to reward your achievements, no matter how small. You could buy that book, album or film you want, maybe give yourself some 'me' time or go on a trip. But try to avoid unhealthy treats that might have a negative impact on other goals. Use the activity chart included with this guide to record your achievements, and encourage your family and friends to do the same.

Remember, you can ask for advice from your cancer specialist or GP if you have any questions about becoming more active. They may refer you to an exercise specialist or physiotherapist.

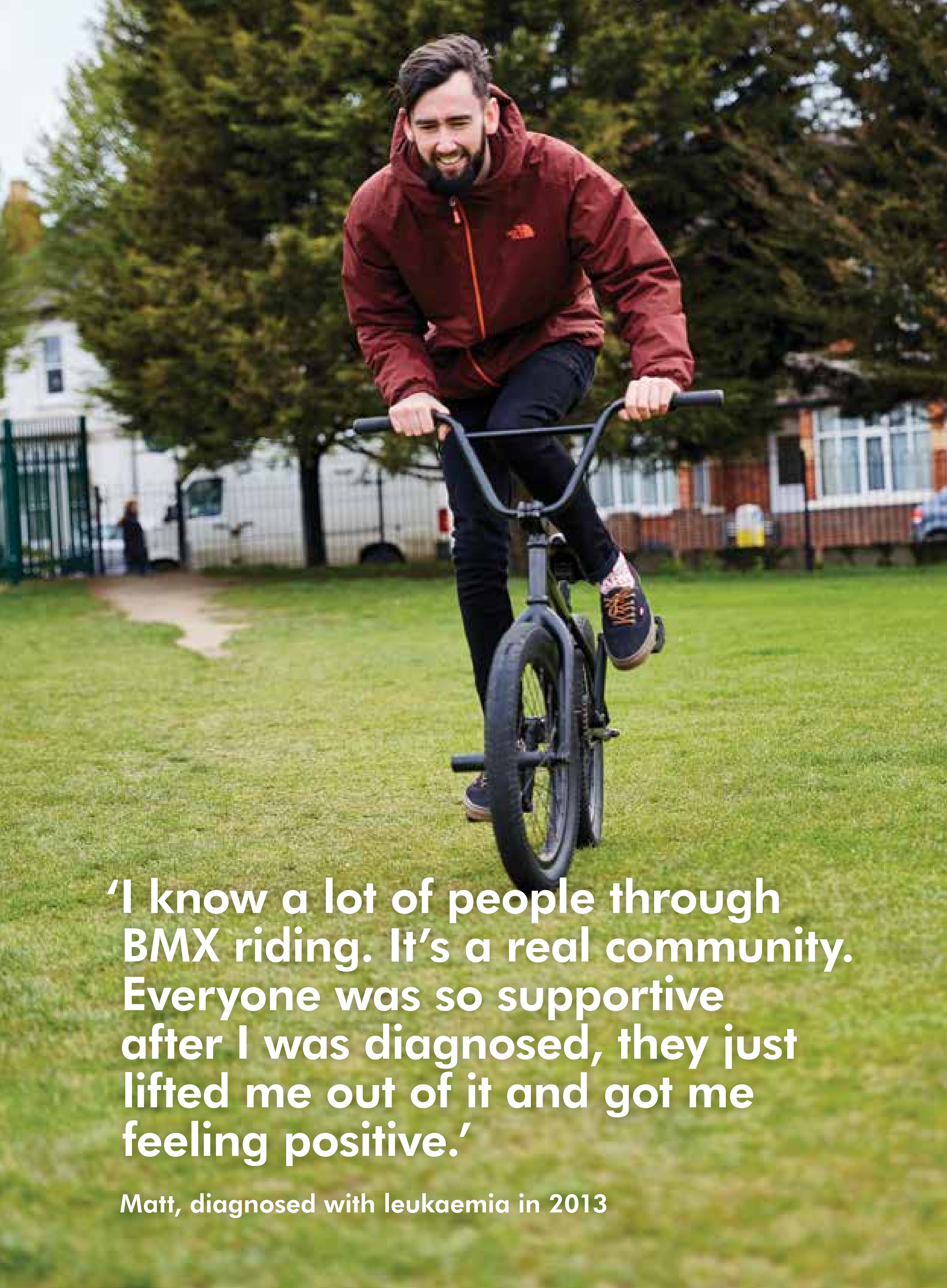

'I know a lot of people through BMX riding. It's a real community. Everyone was so supportive after I was diagnosed, they just lifted me out of it and got me feeling positive.'

Matt, diagnosed with leukaemia in 2013

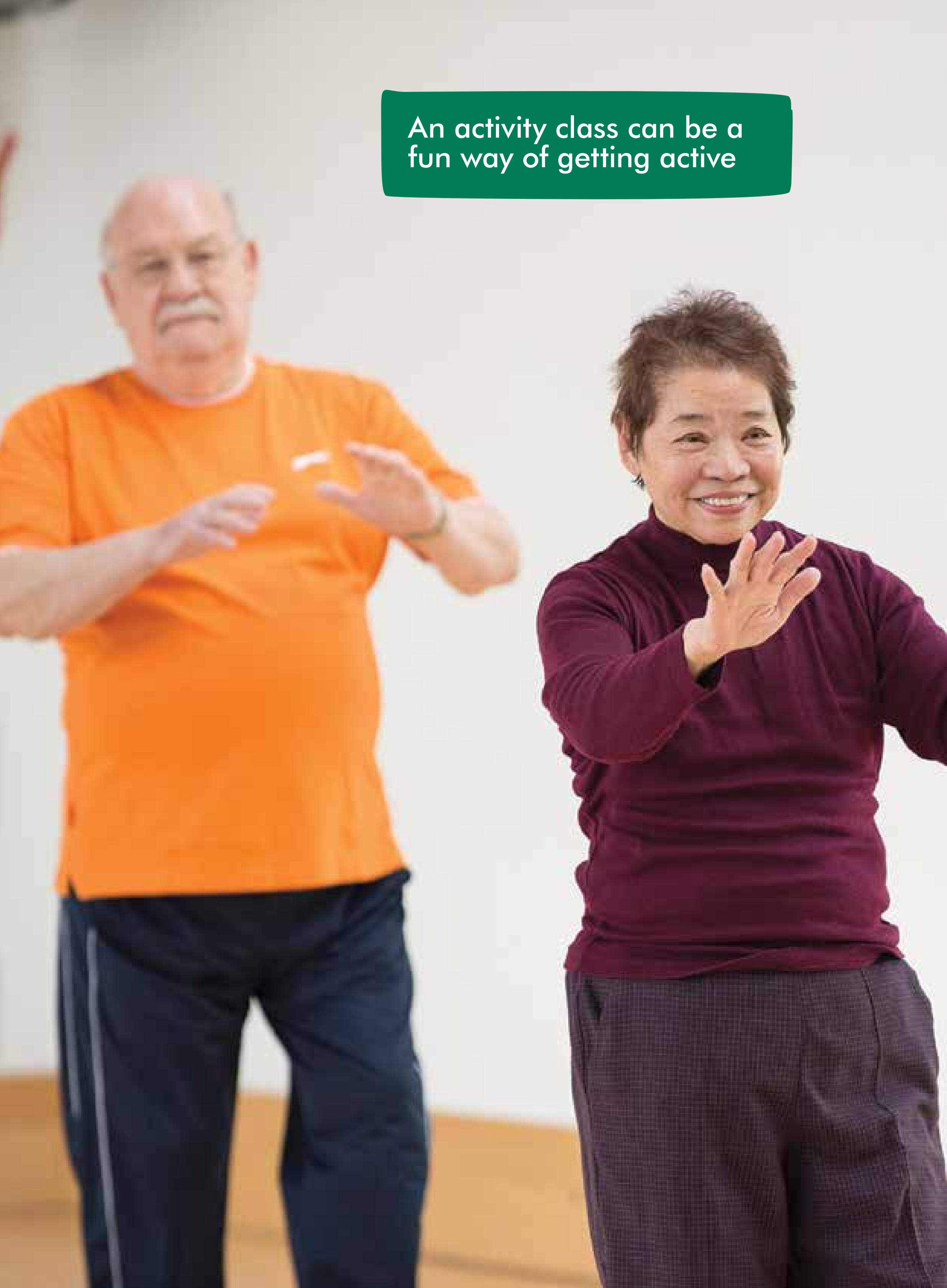

An activity class can be a fun way of getting active

SETTING GOALS AND STAYING ACTIVE

How to set goals 24

Tracking your progress 30

Action plan 34

Next steps 36

My activity diary 39

How to set goals

It's important you set goals that are achievable for you. Start at a level that suits you and don't try to do too much too soon. Find an activity you like and then gradually build up how long you do it for and how often. Build up at your own pace. For example, if you enjoy walking, start by aiming for 10 minutes and then try slowly building up the length and speed of your walks. You could also try going a little more often.

How you feel may change from day to day, so listen to your body to work out what types of activity feel right for you.

Your future goal

You may find it helpful to set yourself a target for a few weeks or months into the future. Think about your interests, your current level of activity, the type of cancer you have and your treatment.

Here are two examples of future goals:

'I will join my local walking group so that in three weeks' time I can get to the shops on my own.'

'I want to find a class at the leisure centre for people with long-term conditions. I want my arms to be strong enough to lift my grand-daughter at Christmas.'

Encourage your family and friends to join you, and try to co-ordinate your goals so you can help and support each other. You might find it helpful to look back at what you wrote in the table on page 11. If this is something that will work for you, try answering the following question:

What would you like to achieve from being more active, and how would you like to feel in a few weeks or months?

Now try writing your future goal:

____

____

____

Now you have a future goal, you can start to set yourself some short-term goals to help you work towards this.

Your short-term goals

Gradually build up the amount you do to help you achieve your goal. This can help you stay motivated when you're getting started. Build up to a comfortable level and go at your own pace. If you are going through treatment, you might only be able to do a certain level of activity. Try to work around your treatment by making the most of your good days and resting when you need to.

Here are two examples of short-term goals that could help you work towards a future goal:

'On Monday at 10am, I will walk in the park for 10 minutes with a friend.'

'On weekdays, I will stand and walk on the spot for 5 minutes every hour with my wife.'

Try not to set too many goals at once. Remember to start slowly and at a level that is right for you. Encourage your family and friends to do the same, so you can get active together.

It's a good idea to make your goals as specific as possible. Where you can, try to include:

- when you will do the activity
- what activity you will do and for how long
- who you will do the activity with.

Try writing your first short-term goal now:

You can set more short-term goals each week in the activity diary on pages 40–54.

'You don't have to go and climb a mountain. To get to the top of your stairs can be a huge challenge if you're having chemotherapy, but if you can do that, then maybe you can do it twice the next day.'

Michelle, diagnosed with thyroid cancer in 2003

Overcoming barriers

It can be helpful to think in advance about what could get in the way of you becoming and staying more active. This could be the weather, working late, feeling unwell or not being motivated. If you have bladder or bowel problems, you might be worried about finding a toilet quickly.

Think about how you could overcome these barriers to being active. For example, doing an indoor activity instead, joining a walking group or exercise class, or going with a relative or friend.

If your treatment is affecting how your bowel or bladder works and you are worried about toilet access while getting active, our toilet card could help. Visit **be.macmillan.org.uk** and search for 'toilet card'.

Look back at the short-term goal you have just set on page 27. What might get in the way of you achieving it? What could you do to avoid those barriers? You could have an indoor back-up plan in case the weather is bad. Or if you think you might find it hard to motivate yourself, arrange to meet a friend there or go with your family.

For example:

'On Monday at 10am, I will walk in the park for 10 minutes with a friend. **If something gets in the way, I will do the Move more DVD at home.'**

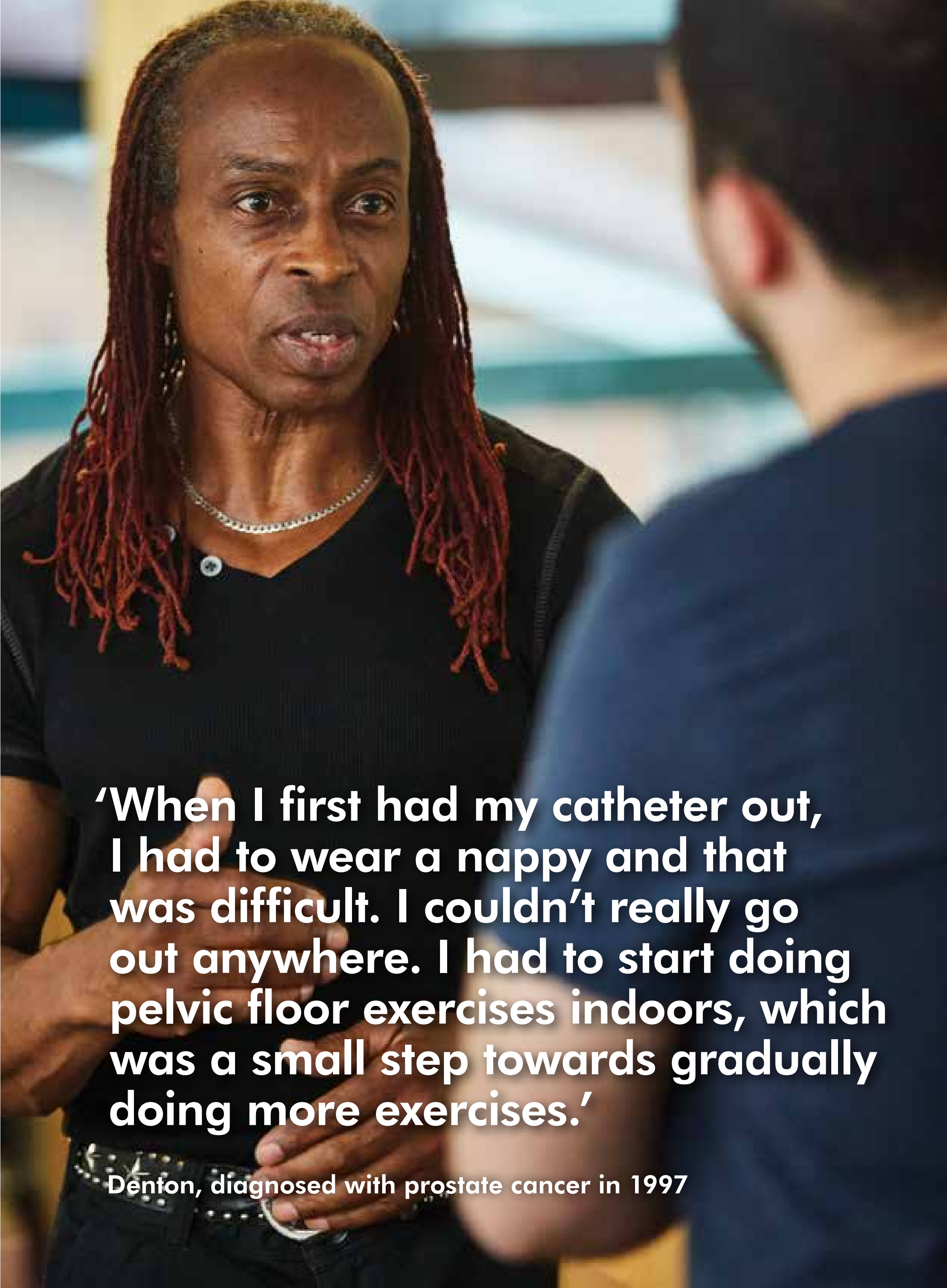

‘When I first had my catheter out, I had to wear a nappy and that was difficult. I couldn’t really go out anywhere. I had to start doing pelvic floor exercises indoors, which was a small step towards gradually doing more exercises.’

Denton, diagnosed with prostate cancer in 1997

Tracking your progress

The activity diary on pages 40–54 will help you track your progress. You can use it to set yourself some realistic and achievable short-term goals each week. There is space to record your activity for 12 weeks.

Using the activity diary

Use the diary to set goals each week, and to record the activity you do and how it made you feel. Using the diary will also help you see whether you've set the right goals. You can change them if they are too challenging, or not challenging enough. The following examples show you how to use the diary.

My goal this week:

On **Monday** at **10am** I will **walk** in the park for **10 minutes** with a **friend**. If something gets in the way, I will do the **Move more DVD** at home.

When	What	Who with	How long	How I felt after
Monday 10am	Walk	Alexis	10 mins	Refreshed, slept much better

My goal this week:

On **weekdays** I will **walk on the spot** for **5 minutes every hour** with my **wife**. If something gets in the way, I will do some **stretches** instead.

When	What	Who with	How long	How I felt after
Monday every hour	Walk on spot	Emma	5 mins	Like I'm taking control

You might prefer to record your activity in the calendar on your smart phone, if you have one.

Rewarding yourself

It is important to reward your achievements, no matter how big or small. Think of something you enjoy, and reward yourself when you achieve your goal. There's space in the activity diary to write how you will reward yourself each week. For example:

I will reward myself by going to the cinema to see a film.

If you don't achieve your goal, don't be disheartened. This is an opportunity to have another look at your short-term goals and set a new goal that you can achieve.

Using the activity chart

We have included an activity chart (see photograph below) in the front pocket of this guide. You could put this chart somewhere you can see it easily and record how you're doing each day. The chart has space for 12 weeks of activity too.

We've also included a magnet, in case you would like to put the chart on your fridge.

Your family and friends could record their activities on it too.

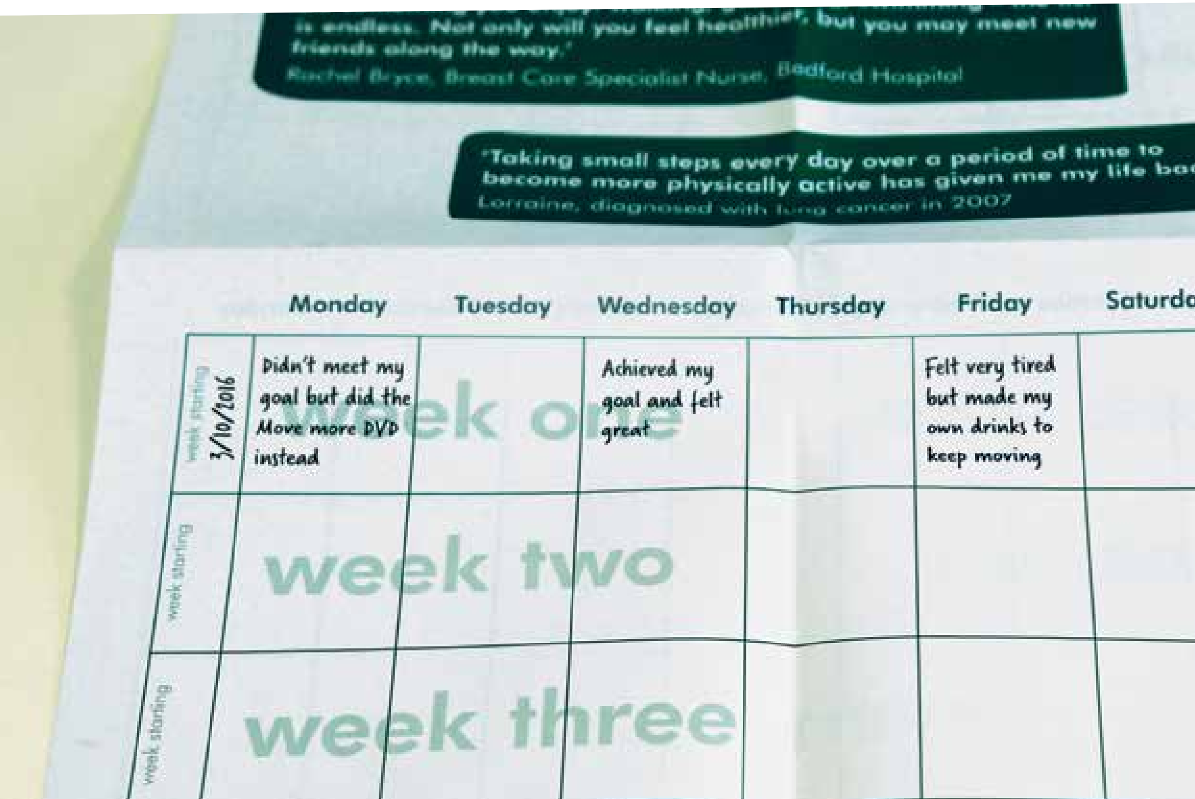

> ‘You don’t have to run a marathon, you don’t have to lift heavy weights, you don’t even have to sweat. Exercise is a great way to build up energy levels and strength.’
>
> **Pete, diagnosed with head and neck cancer in 2011**

For more encouragement and ideas about getting active, visit our website at **macmillan.org.uk/beactive**

Here you can:

- sign up to our regular newsletters for more advice and tips about becoming active
- find activities in your local area
- ask our experts questions about being active
- join our online community to share your experiences and meet others who have used this guide to become more active.

You can also call the Macmillan Support Line free on **0808 808 00 00** (Monday–Friday, 9am–8pm) for more information and support.

Action plan

Some people find it useful to summarise their reasons for wanting to move more and make an action plan to help make it happen. Looking back at this as you begin to get active can help keep you motivated.

Try to fill in the gaps below with your plan for moving more. Your family or friends can write down their plans too, so you can keep each other motivated.

It might help you to look back at what you wrote in the 'Getting started' section (pages 9–21).

I want to move more because:

____

____

To do this, I am going to:

____

____

I will do this with:

Things that might get in my way are:

I will overcome these barriers by:

I will reward myself with:

Why not share your plan with other **Move more** guide users on our online community? Your story could inspire others who are trying to be more active. Visit **macmillan.org.uk/beactive** for more information.

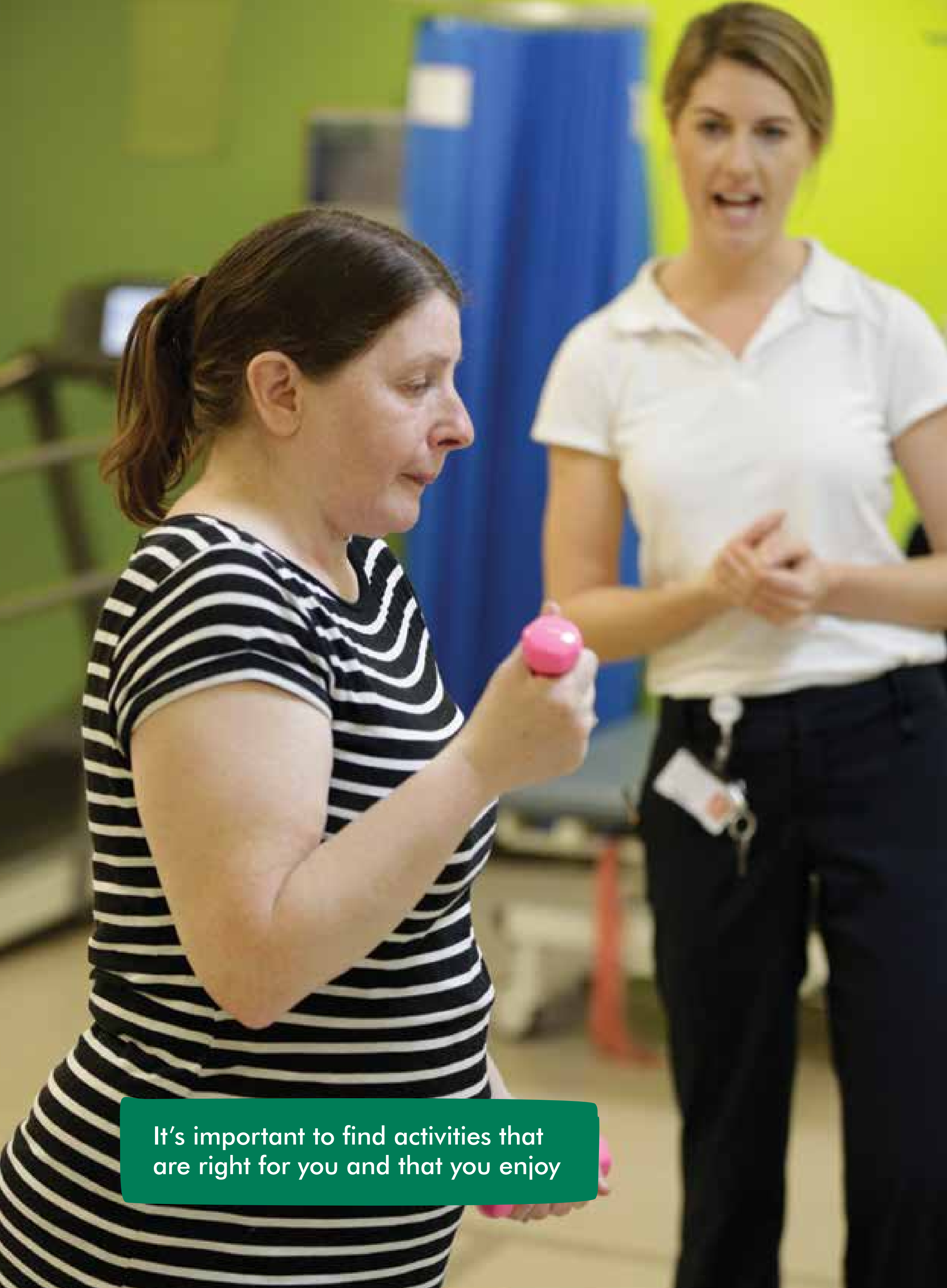

It's important to find activities that are right for you and that you enjoy

Next steps

When you're ready, start to set some weekly goals and complete the activity diary on pages 40–54. You could also use the activity chart (in the front pocket of this guide) to track your achievements and share your progress with family and friends.

Many people with cancer tell us that once they get started, they find that being active becomes an enjoyable part of their lives. Most people begin to notice the benefits quickly, including feeling less tired and less stressed, and having more confidence. Making small changes and knowing the benefits to your health can keep you going, even on days when it's hard to feel motivated.

If you're struggling, don't be hard on yourself. Just remind yourself of all the benefits and get started again. Good luck!

If you have any questions, ask your cancer specialists or GP. You can also visit **macmillan.org.uk/beactive** and join in with an ask the expert session to get more advice. From here, you can also sign up to our newsletter or visit our online community to chat with people like you who are getting active.

If you have any more questions, need support or just want to chat, you can call the Macmillan Support Line free on **0808 808 00 00** (open Monday–Friday, 9am–8pm).

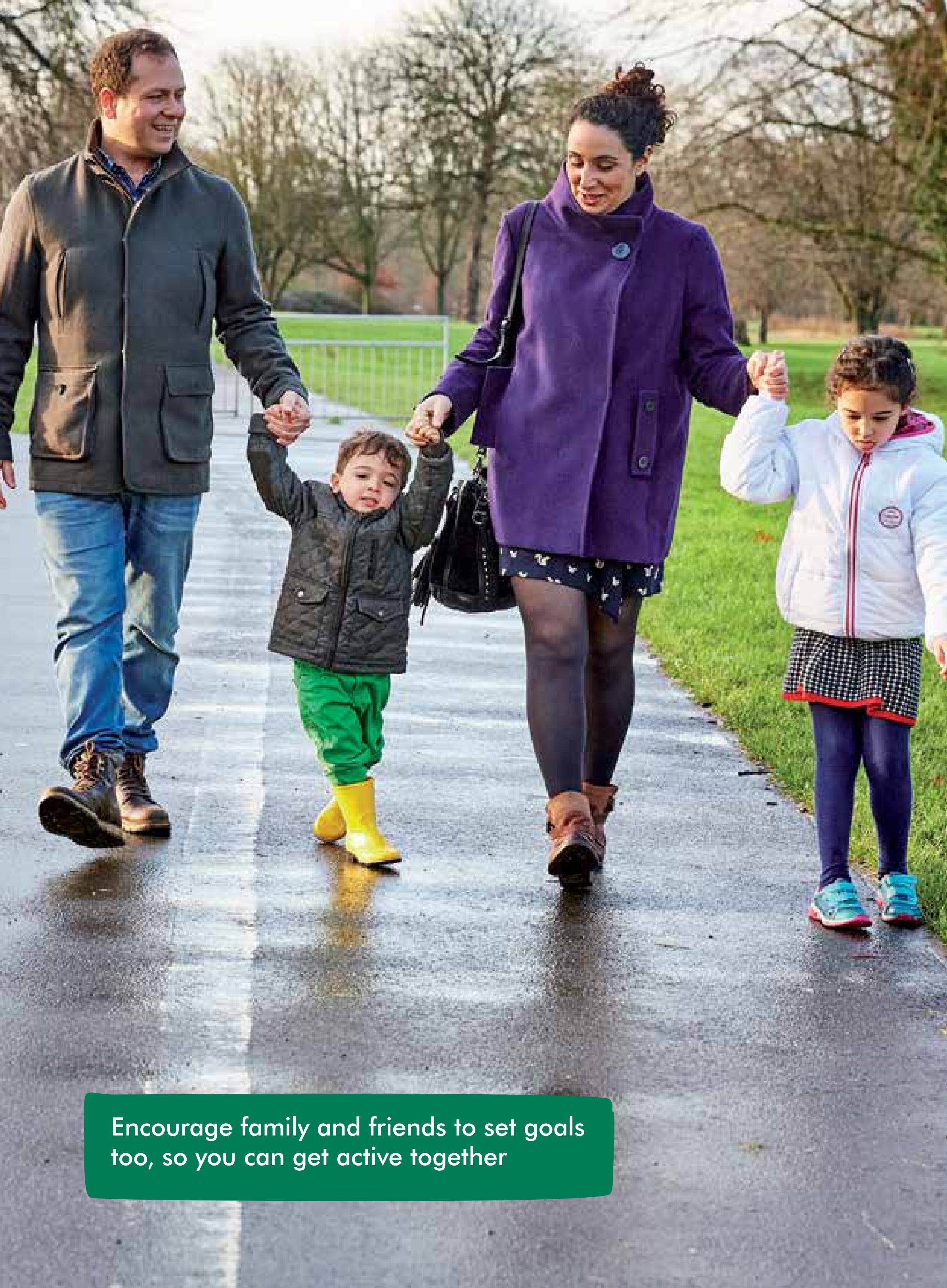

Encourage family and friends to set goals too, so you can get active together

My activity diary

Over the next few pages you will find a 12-week activity diary, where you can start to put into practice everything you have learnt from the guide so far. Here you can record:

- your goals for becoming active
- what might get in the way of you achieving your goal
- how you will overcome those barriers
- how you will reward your achievements
- the details of your activity and how you felt after doing it.

'Setting myself little, achievable goals and taking small steps everyday over a period of time to become more active has given me my life back.'

Lorraine, diagnosed with lung cancer in 2007

WEEK 1

Date:

My goals this week

1.

2.

3.

I will reward myself by ______________________________

When	What	Who with	How long	How I felt after

Remember you can also record your achievements on your activity chart in the front of this guide.

WEEK 2

Date:

My goals this week

1.

2.

3.

I will reward myself by ______________________________

When	What	Who with	How long	How I felt after

Remember you can also record your achievements on your activity chart in the front of this guide.

WEEK 3

Date:

My goals this week

1.

2.

3.

I will reward myself by ______________________________

When	What	Who with	How long	How I felt after

Remember you can also record your achievements on your activity chart in the front of this guide.

WEEK 4

Date:

My goals this week

1.

2.

3.

I will reward myself by ______________________

When	What	Who with	How long	How I felt after

Remember you can also record your achievements on your activity chart in the front of this guide.

WEEK 5

Date:

My goals this week

1.

2.

3.

I will reward myself by ____________________

When	What	Who with	How long	How I felt after

Remember you can also record your achievements on your activity chart in the front of this guide.

WEEK 5

Turn back to the start of this diary. Is there a difference in what you can do now, compared to what you could do then?

Make a note of any changes, particularly in how you feel:

WEEK 6

Date:

My goals this week

1.

2.

3.

I will reward myself by ____________________

When	What	Who with	How long	How I felt after

Remember you can also record your achievements on your activity chart in the front of this guide.

Look at the future goal you wrote on page 25. Are you getting nearer to achieving it? Maybe you've already achieved it, or it's no longer what you want to work towards. If that's the case, you can always set a new one now.

If you need to, use the space below to write your new future goal:

WEEK 7

Date:

My goals this week

1.

2.

3.

I will reward myself by ____________________

When	What	Who with	How long	How I felt after

Remember you can also record your achievements on your activity chart in the front of this guide.

WEEK 8

Date:

My goals this week

1.

2.

3.

I will reward myself by ______________________________

When	What	Who with	How long	How I felt after

Remember you can also record your achievements on your activity chart in the front of this guide.

WEEK 9

Date:

My goals this week

1.

2.

3.

I will reward myself by ______________________________

When	What	Who with	How long	How I felt after

Remember you can also record your achievements on your activity chart in the front of this guide.

WEEK 10

Date:

My goals this week

1.

2.

3.

I will reward myself by ______________________________

When	What	Who with	How long	How I felt after

Remember you can also record your achievements on your activity chart in the front of this guide.

WEEK 11

Date:

My goals this week

1.

2.

3.

I will reward myself by ________________________________

When	What	Who with	How long	How I felt after

Remember you can also record your achievements on your activity chart in the front of this guide.

Turn back to the start of this diary – is there a difference in what you can do now, compared to what you could do then? Is there a difference in how you feel too?

Make a note of any changes here:

WEEK 12

Date:

My goals this week

1.

2.

3.

I will reward myself by ____________________

When	What	Who with	How long	How I felt after

Remember you can also record your achievements on your activity chart in the front of this guide.

Congratulations on being active for 12 weeks!

For lots more support and information, visit our website at **macmillan.org.uk/beactive** You can also call our cancer support specialists for more information on **0808 808 00 00**.

'Regular physical activity is a key part of cancer survivorship – a good way of keeping fit, and maintaining a healthy weight and good mental health. Add in the social aspects, along with the sense of pride and satisfaction when you achieve your targets – what other motivation is needed? So get moving!'

Carole, diagnosed with pancreatic cancer in 2009

Disclaimer

We make every effort to ensure that the information we provide is accurate and up to date but it should not be relied upon as a substitute for specialist professional advice tailored to your situation. So far as is permitted by law, Macmillan does not accept liability in relation to the use of any information contained in this publication, or third-party information or websites included or referred to in it.

Thanks

This guide has been written, revised and edited by Macmillan's Cancer Information Development team. It has been approved by our Chief Medical Editor, Tim Iveson, Macmillan Consultant Medical Oncologist.

With thanks to: Dr Tim Anstiss, Director, Academy for Health Coaching; Dr Melvyn Hillsdon, Associate Professor of Exercise and Health Behaviour, Sport and Health Sciences, Exeter University; Dr Heather McKee, Lecturer in Health and Exercise Psychology (Behaviour Change), St Mary's University; Dr Ollie Minton, Macmillan Consultant and Honorary Senior Lecturer in Palliative Medicine, St George's University Hospitals NHS Foundation Trust; Dr Clare Stevinson, Lecturer in Behavioural Aspects of Physical Activity and Health, Loughborough University; Justin Webb, Physical Activity Engagement Manager, Macmillan Cancer Support. Thanks also to the people affected by cancer who reviewed this guide, and those who shared their stories.

Sources

We've listed a sample of the sources used in this publication below. If you'd like further information about the sources we use, please contact us at **bookletfeedback@macmillan.org.uk**

BBC Get Inspired. www.bbc.co.uk/sport/get-inspired (accessed February 2016).
Thomas, et al. Physical activity after cancer: an evidence review of the international literature. British journal of medical practitioners. 7(1) a708. 2014.
Webb. Developing a health promotion campaign to influence physical activity in people living with cancer. Unpublished manuscript. University of Hertfordshire, UK. 2016.

When you have cancer, you don't just worry about what will happen to your body, you worry about what will happen to your life. Whether it's concerns about who you can talk to, planning for the extra costs or what to do about work, at Macmillan we understand how a cancer diagnosis can affect everything.

No one should face cancer alone. So when you need someone to turn to, we're here. Right from the moment you're diagnosed, through your treatment and beyond, we're a constant source of support, giving you the energy and inspiration to help you take back control of your life.

For support, information or if you just want to chat, call us free on **0808 808 00 00** (Monday to Friday, 9am–8pm) or visit **macmillan.org.uk**

Hard of hearing? Use textphone **0808 808 0121**, or Text Relay.
Non-English speaker? Interpreters available.
Braille and large print versions on request.

Move more.
Be a more active you.

© Macmillan Cancer Support, July 2016. 2nd edition. MAC13314.
Next planned review 2019. Macmillan Cancer Support, registered charity in England and Wales (261017), Scotland (SC039907) and the Isle of Man (604). Printed using sustainable material. Please recycle.

Health & care information you can trust
The Information Standard Certified Member
