# Supplementary material for: The Effect of the Move More Pack on the Physical Activity of Cancer Survivors: Protocol for a Randomized Waiting List Control Trial with Process Evaluation
Source: JMIR Res Protoc. 2017 Nov 9;6(11):e220. doi: 10.2196/resprot.7755 (PMC5701086; doi:10.2196/resprot.7755)
Supplement: Multimedia Appendix 2 [file resprot_v6i11e220_app2.pdf]

WE ARE MACMILLAN. CANCER SUPPORT

A practical guide to living with and after cancer

PHYSICAL ACTIVITY AND CANCER

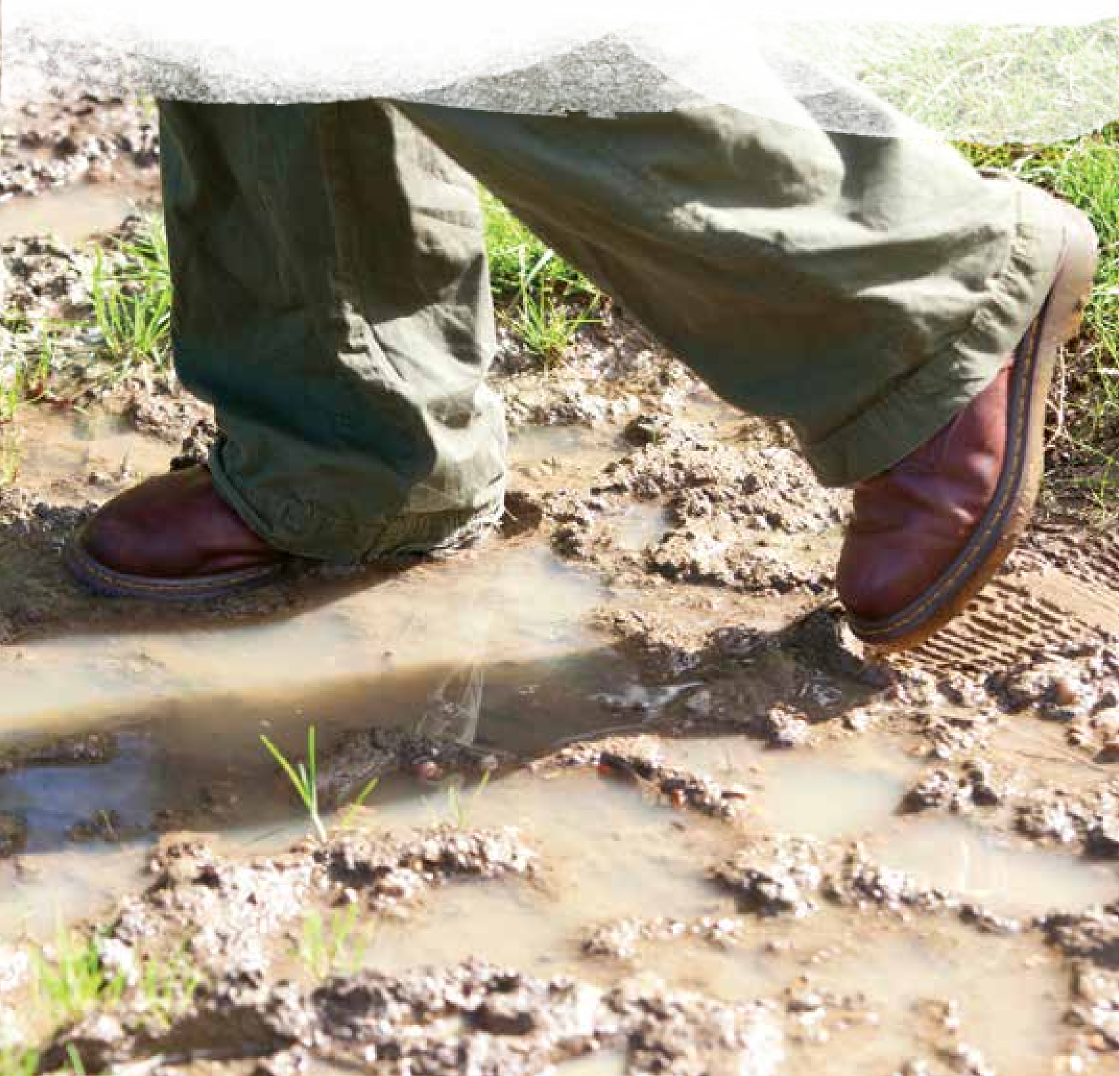

About this booklet

Many people affected by cancer want to make positive changes to their lives. Taking steps to have a healthier lifestyle can be a big part of this.

This booklet tells you about the benefits of being physically active during and after cancer treatment. It gives practical advice on how to get started. Physical activity is any form of exercise or activity involving movement that uses your muscles. This includes lots of everyday activities, such as walking, housework and gardening. Any sort of activity helps maintain or improve your fitness, health and well-being. Making small changes and getting up to move around more can help you before, during and after cancer treatment.

We've included comments from people affected by cancer. Some are from people who've chosen to share their story with us. To share your story, visit **macmillan.org.uk/cancervoices** Other comments are from the website **healthtalk.org**

This booklet contains contact details for other organisations that can help (see pages 52–55). There are also ways to find activities near you (see pages 42–45).

If you'd like to discuss this information, call the Macmillan Support Line free on **0808 808 00 00**, Monday–Friday, 9am–8pm. If you're hard of hearing you can use textphone **0808 808 0121**, or Text Relay. For non-English speakers, interpreters are available. Alternatively, visit **macmillan.org.uk**

If you find this booklet helpful, you could give it to your family and friends. They may also want information to help them support you.

We have another resource that you may find helpful, which is called **Move more: your guide to becoming more active**. It includes:

- a motivational guide to help you get started
- information on setting physical activity goals
- simple ways to be more active
- examples of what other people have done
- an activity diary
- an activity DVD with exercises you can do.

You can order the **Move more** guide from **be.macmillan.org.uk** or by calling our support line on **0808 808 00 00**.

For more information and support about getting active, visit **macmillan.org.uk/beactive**

Contents

Why be more active?	4
Being active before treatment	7
Being active during treatment	8
Reducing side effects	10
Being active after treatment	14
Advanced cancer and physical activity	18
Being safe	19
How much activity is recommended?	20
How much activity is right for you?	23
What types of activity should you do?	24
Who can help?	36
Getting started	40
What activities are available near you?	42
Next steps	46
About our information	47
Other ways we can help you	49
Other useful organisations	52

Why be more active?

When you are living with or after cancer, becoming more active can be a positive change to make in your life. Evidence shows that physical activity can benefit people affected by cancer in several different ways. Research suggests that being physically active, along with eating a healthy diet, can help reduce the risk of recurrence for some cancer types and increase survival. It also helps reduce the risk of developing other health problems, such as heart disease, stroke and diabetes.

Being active before, during and after treatment can:

- reduce tiredness (fatigue)
- improve your quality of life
- help look after your heart
- reduce anxiety and depression
- help you maintain a healthy weight
- strengthen your muscles, joints and bones
- improve your flexibility and help keep you supple
- increase your confidence.

At first, you might be nervous about starting and building up your activity, especially if you haven't been active for a while. You may worry that you are too tired, don't know how to start or don't know what is best for you to do. You may also be concerned about injuring yourself. But even a little physical activity is better than none at all. It can help you feel less stressed and lift your spirits if you're feeling low. It will also help you feel more in control, because you are doing something positive for yourself.

Before treatment

If you know you are going to have surgery, your doctor might encourage you to start some physical activity before your operation. This may help with your recovery.

See page 7 for more information about physical activity before treatment.

During treatment

During treatment, it is best to avoid sitting or lying for long periods in the daytime. It is a good idea to do some gentle activity, such as short walks throughout the day. During treatment you may be tired and think that exercise will make this worse. Your family and friends may advise you to 'take it easy'. But studies have shown that if you are not active, you may feel more tired and lose muscle strength and stamina. Physical stamina is how well your heart and lungs cope with walking quickly or running for a few minutes.

Regular activity will reduce the risk of blood clots (thrombosis). These are more common after cancer, especially if you:

- have recently had surgery, chemotherapy or a hormonal therapy
- spend a lot of time not moving.

There is more information about how physical activity can help you during cancer treatment on pages 8–9.

After treatment

After treatment, being physically active can help you cope with and recover from some side effects. There is more information about this on pages 14–17.

What is physical activity?

Being physically active can mean doing simple daily activities, such as:

- light housework, for example vacuuming
- light gardening
- walking to the shops
- taking the stairs instead of the lift.

Or it can mean more energetic activities, such as:

- dancing
- running
- cycling
- badminton
- digging in the garden
- going to the gym.

What you choose to do will depend on how your treatment is going, which activities you enjoy, and your level of fitness. This will also affect the amount of activity you do.

'When so many things are going wrong, it feels great to do something that you can tell makes you feel better as you do it.'

Ailsa

Being active before treatment

If you know you are going to have surgery, your doctor might encourage you to start some physical activity before the operation. This is to help improve your general fitness level. It can also help with your recovery after surgery.

Being active before any treatment starts can help prepare your body for treatment. This may mean you have fewer side effects, or that they're less severe. It can also help you feel better in yourself. And it may help you recover more quickly.

Some people have to start treatment straight away. But if you don't, physical activity will help you prepare for future treatment.

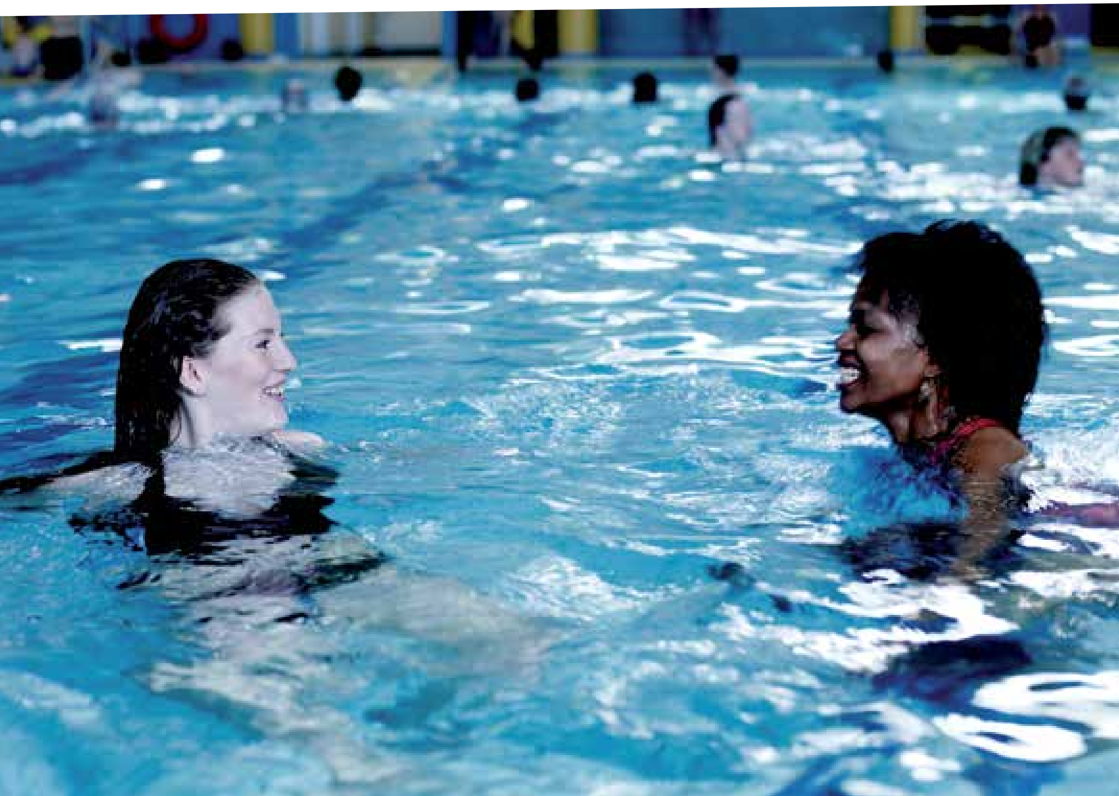

Being active during treatment

Being physically active during treatment is generally very safe. You will usually know how much activity you can manage, but there may be activities you need to avoid or be careful with. Try to make steady improvements every month, as long as your treatment allows. There may be some weeks when you have to do less, such as immediately after chemotherapy. And there may be some weeks when you can do more. The important thing is not to suddenly do intense exercise you are not used to. This can cause muscle strain.

It's best to focus on increasing the amount of activity you do in the long term. Try not to worry about any short-term setbacks. Any physical activity you can do during treatment will help you maintain your fitness.

If you are not sure what to do, ask a cancer exercise professional. You may have one available at the hospital, or your GP may be able to refer you to one in a local leisure centre. In some areas, you can get individual exercise programmes. You could also ask your cancer doctor or specialist nurse for exercise advice. There is more information about who can help you start being more active on pages 36–39.

'I didn't run during my treatment, as I didn't feel I should. I tried it once and got light-headed, so I walked instead. It got fresh air in my lungs and made me feel better.'

Sharon

What you can manage will depend on your level of fitness and the treatment you are having. You might just be trying to reduce the amount of time you spend sitting down. You could try doing some light housework, making yourself a snack or going for a walk with family or friends. Remember to pace yourself. Try not to do too much, even on a good day.

If you already exercised before treatment, you may find you need to do it at a lower intensity during treatment – see page 25 for more information. You can gradually increase it again after treatment ends.

Studies have shown that exercising during chemotherapy helped people stay active or feel better about themselves.

‘The feel-good factor I got after exercise was fantastic. The focus of exercise during and after treatment has gone a long way to helping me get to where I am now, both physically and mentally.’

Michaela

Reducing side effects

You may have side effects during your treatment and for some months or years after it finishes. Being more physically active may help reduce some of these.

Reduce tiredness

Feeling like you have no energy is a very common side effect of treatment. It can sometimes last for months after treatment ends. Being more active can help you manage fatigue. Even doing a little bit more activity can help improve your energy levels.

'I found taking a walk in the park on sunny days during chemo treatment made me feel so much happier and ready to take on the world again.'

Namdev

For most people, a good way to be active is to go for short walks every day, even for a few minutes to start with. Walk at a comfortable pace for you. Don't push yourself too hard. Doing too much, too soon, may make you more tired.

Simple strength exercises can also be helpful when your energy levels are low. These exercises include 'sit to stand' (see page 26), tai chi and qi gong (see page 27).

Improve your mood and reduce stress

Stress, anxiety and low mood are common problems during and after treatment. Physical activity encourages the brain to produce chemicals (endorphins) that improve mood and reduce stress.

Being active with other people can really help. You could exercise with family or friends. Or you could join a cancer rehabilitation programme and exercise with other people who understand what you are going through. It can also help to be active outdoors somewhere green, such as a park. You could try gardening or joining a walking group (see page 43).

‘Following diagnosis, I became really depressed. Exercise was part of the cure – it really worked and still does.’

Aleksander

Sleep better

Being more active during the day helps you relax and sleep better at night.

Build muscle strength

You may lose muscle bulk and strength during and after treatment. This happens when your muscles aren't being used as much as usual. It's also a side effect of hormonal therapy for prostate cancer or of steroid treatment.

Strength training will help increase your muscle strength. This includes moving from sitting to standing, climbing stairs and resistance training using light weights or bottles of water. You can gradually build this up after treatment. See page 26 for more information about strength training.

Ease joint pain and improve flexibility

If you have painful joints, regular physical activity can help ease the pain by building muscle strength and improving flexibility. Joints that can move flexibly are less likely to be sore. There is more information about flexibility exercises on page 27.

If you have sore joints, weight-supported exercises are good. These include swimming and cycling. They are good because your weight is supported and there is less strain on the joints in your legs and back. Weight-supported exercises can help you avoid stiffening up. You can do them even when you're feeling too tired to do much else.

'Physical activity aided my emotional well-being and helped me remain positive. I used to come back from the doctor's and do a dance class with the children. I would forget about everything. It gave me time to not dwell on the negative.'

Libby

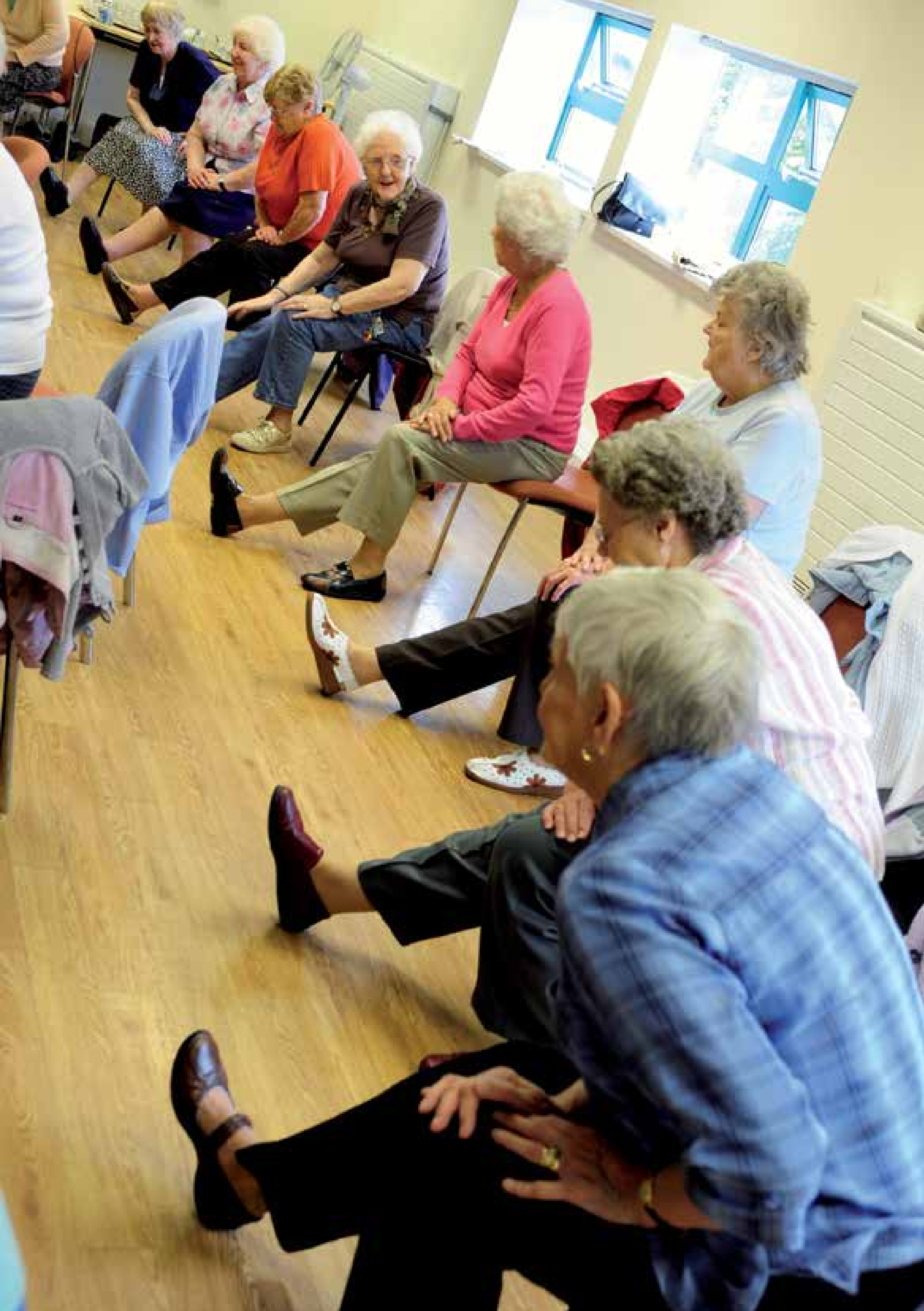

Being active after treatment

Being physically active after treatment is a positive step in your recovery. It may help reduce the risk of certain cancers coming back. It may also help you manage and reduce the risk of:

- late effects of treatment such as tiredness
- weight gain
- other health problems.

Physical activity and late effects

These are side effects that may develop months or years after treatment ends.

Heart health

Some treatments may slightly increase the risk of heart problems in the future. These include radiotherapy that's given close to the heart and certain chemotherapy or targeted therapy drugs.

Aerobic activities (see pages 24–25) can help protect your heart and reduce the risk of late effects developing. Brisk walking, running, skipping, cycling, dancing and swimming are all aerobic activities.

Our booklet **Heart health and cancer treatment** has more information about looking after your heart.

Bone health

Hormonal therapies for breast and prostate cancer can increase the risk of bone thinning (osteoporosis). So can having an early menopause due to cancer treatments.

Activities where you are supporting your own body weight will help keep bones strong. These are sometime called weight-bearing exercises. They include walking, dancing and resistance training (see page 26).

If you have osteoporosis, get advice on exercise from your doctor, nurse, physiotherapist or exercise specialist. For more information on being safe if you have bone problems, see page 32.

Our booklet **Bone health** has more information about looking after your bones.

Confidence

Most people feel overwhelmed when they are told they have cancer, and have many different emotions during and after treatment. Different people have different feelings, but they may include fear, uncertainty, anxiety, isolation and depression.

Research has shown that being physically active during treatment can help improve your mood and confidence.

'Obviously I had to rest after treatment, but I built up my movement slowly. It took about three months to get back to the level of fitness I had before.'

Libby

Keeping to a healthy weight

People often don't expect to gain weight while they are having cancer treatment, but it can happen. There can be several reasons for this. Treatment can make you tired, so you may be less active than usual. Hormonal therapies and steroids can also cause weight gain.

Being active and eating healthily are major factors in managing your weight. Keeping to a healthy weight can help reduce the risk of:

- joint problems
- back problems
- developing a new (primary) cancer – excess body fat has been linked to some bowel, breast and womb cancers.

We have a booklet called **Managing your weight after cancer treatment** that we can send you.

Other health problems

After cancer treatment, some people are more at risk of developing other health problems. Being physically active can help you manage or reduce your risk of:

- high blood pressure
- stroke
- type 2 diabetes
- kidney disease
- depression
- anxiety.

Reducing the risk of cancer coming back

There is some emerging evidence that being physically active at the levels recommended (see pages 20–21) can reduce the risk of certain cancers coming back or progressing.

Research in this area is ongoing. We need more evidence before we can say exactly how much and what specific exercise is needed to get these benefits, and exactly how it may protect against a recurrence of cancer. But so far, the signs are that for certain cancers, doing 150 minutes of physical activity a week can make a difference after treatment.

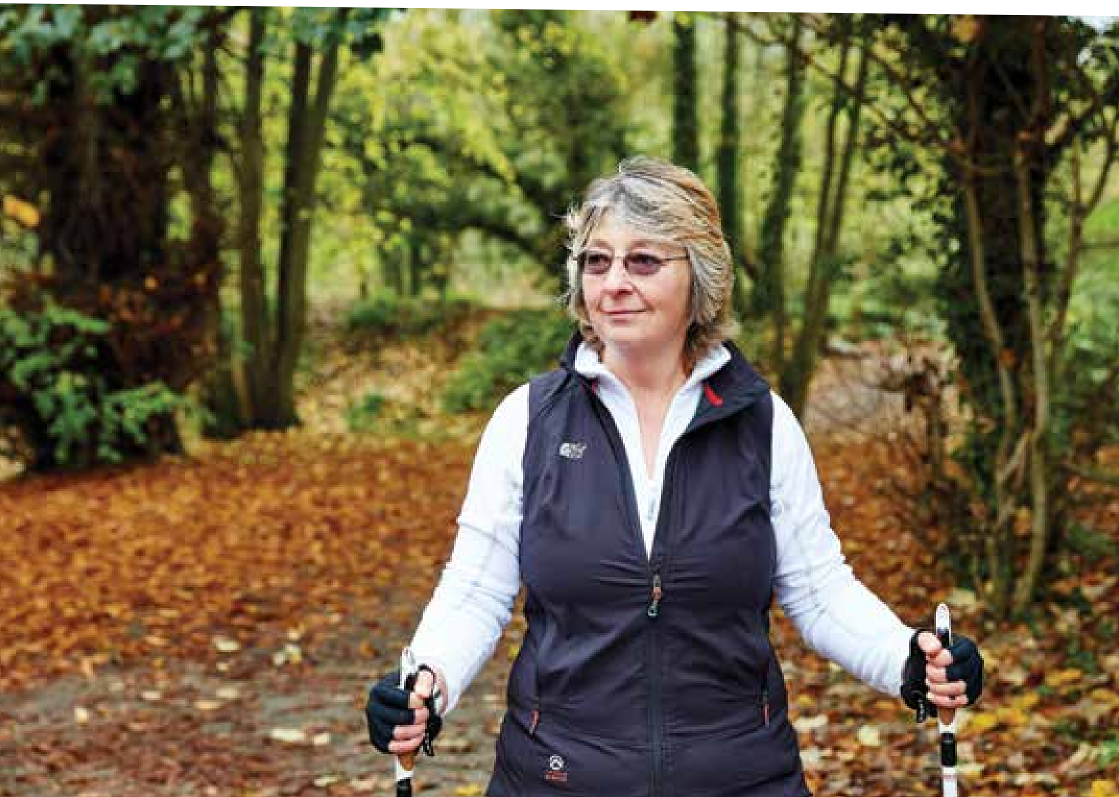

Advanced cancer and physical activity

If you have advanced cancer, being physically active has many benefits. It can help reduce symptoms such as tiredness and poor appetite. Being more active can also help with your walking, balance and confidence.

If you are struggling to do the same amount of physical activity you're used to, you may want to reduce how much you are doing. Or if you currently don't do any physical activity, start slowly and gradually increase the amount you do. To begin with, try to reduce the amount of time you spend sitting or lying down during the daytime. Just moving around the house and doing easy day-to-day things will help.

You may be able to do short walks or gentle stretching exercises (see page 27). You could ask your family or friends to join you.

Resistance exercises can help strengthen your muscles and bones. This helps with getting in and out of chairs or baths, going up and down stairs, and going shopping. It also helps reduce the risk of accidentally falling. There is more information about resistance training on page 26.

You may need to avoid some types of physical activity, for example if the cancer is in your bones or you have bone thinning (see pages 32–24). Ask your doctor or palliative care team for advice before you start. Gentle, low-impact activities include walking, swimming and cycling.

Being safe

Being physically active is safer than being inactive. But it's important to know how to take care of yourself when you start to be more active.

General advice

- Start slowly and gradually build up.
- Don't exercise if you feel unwell, have an infection or a high temperature, or have any symptoms that worry you.
- Stop if you get any sudden symptoms, including feeling dizzy, chest pain, a racing heart, breathing problems, feeling sick, unusual back or bone pain, muscle weakness or a headache that doesn't go away. Contact your doctor if you notice any of these, or any other symptoms.
- Avoid uneven surfaces and activities that increase the risk of falling or hurting yourself, especially if you have bone problems.
- Wear well-fitting trainers or walking shoes. Don't risk getting an injury by wearing the wrong shoes. Drink plenty of water so you don't get dehydrated.
- Protect yourself in the sun.
- Have a healthy snack after exercising, such as a banana.

Which activities are best for you depends on the type of cancer you have, your treatments and any other conditions you have. If you are not sure, get advice from a cancer exercise specialist, your doctor or your physiotherapist.

How much activity is recommended?

There are UK recommendations on physical activity and the activities that can help you achieve them. Healthy adults are advised to do one of the following every week:

- at least two and a half hours (150 minutes) of moderate-intensity aerobic activity
- at least an hour and a quarter (75 minutes) of vigorous-intensity aerobic activity
- a combination of both moderate and vigorous aerobic activity.

We explain what moderate and vigorous activity mean on page 25. To do 150 minutes of activity in a week, you could do 30 minutes of activity on five days of the week. You could break this up into 10 minutes of activity, three times a day. It's important to build this up slowly, at a pace that's comfortable for you. There are ideas for aerobic exercises on pages 24–25.

It's also important to do activities that improve muscle strength on at least two days of the week. If you are older or at risk of falling, you are also advised to do activities that improve co-ordination and balance on at least two days of the week. There is more information about exercises that improve strength, balance and flexibility on pages 26–27.

The infographic opposite shows the amount of physical activity recommended for adults. It also suggests ways of doing it.

Physical activity guide for adults

Be active	Build strength	Improve balance
Keep your heart and mind healthy	Strengthen muscles, bones and joints	Reduce your risk of falling
**How often?** **150** minutes of moderate activity a week **or** **75** minutes of vigorous activity a week	**2** days a week	**2** days a week
Walk, Run, Gardening, Sport, Swim, Stairs	Gym, Aerobics, Carry bags	Dance, Tai chi, Bowling

Sit less

TV Sofa Computer

Break up long periods of sitting down to help keep your muscles, bones and joints strong.

There are international guidelines for physical activity and cancer. They were developed by the American College of Sports Medicine. They are supported by the National Cancer Institute and the British Association of Sport and Exercise Science. This is what they advise:

- Physical activity is safe during and after cancer treatment.
- Avoid being inactive.
- Get back to your normal activities as soon as possible after treatment.
- If possible, slowly build up to the recommended physical activity levels (see pages 20–21).

You can find out more about different types of activity and the recommendations for people of different ages from NHS Choices (**nhs.uk**) and the World Health Organisation (**who.int**).

'To get to the top of your stairs can be a huge challenge if you're undergoing chemotherapy. But if you can do that, then maybe you can do it twice the next day.'

Michelle

How much activity is right for you?

What and how much you do will depend on the following:

- How fit you were before you were diagnosed. If you were active before, you may be able to continue with the same activities when you feel able to.
- The type of cancer and treatments you had or are still having. There may be some activities you need to be careful with. See pages 30–34 for more details.
- Any symptoms or treatment side effects you have.
- Your age and any long-term conditions you have, such as heart problems.
- Whether you have bone loss, lymphoedema, peripheral neuropathy or a stoma (see pages 32–34) because of the cancer or its treatment.

During treatment, your energy levels will vary from day to day. The main aim should be to try to spend less time sitting or lying down.

Choose an activity you enjoy and set some realistic goals for yourself. If you feel very tired the day after activity, you may be trying to do too much, too soon. Over time, you'll be able to increase the amount you do.

After treatment ends, increase your activity slowly. As a general rule, and only if you can, try to slowly increase it to the recommended 150 minutes of moderate-intensity activity or 75 minutes of vigorous-intensity activity a week (see pages 20–21).

What types of activity should you do?

There is no one activity that is best for everyone. Choose activities you enjoy and that fit in with your life. If possible, do a mix of activities that improve your aerobic fitness, strength and flexibility.

Aerobic exercise

Aerobic exercise works your heart and lungs and uses large muscle groups, such as those in your legs. It does this repetitively for a period of time. It makes you breathe harder and raises your heart rate, so your heart works harder to pump blood through the body. It's particularly good for your heart and cardiovascular system. These are common aerobic exercises:

- **Daily activities.** Taking the stairs, doing housework, gardening, walking a dog and playing games with children are all considered moderate-intensity activities by the World Health Organisation.
- **Walking briskly.** This is one of the simplest and most effective aerobic exercises. It is also a weight-bearing exercise, because your feet and legs support your body's weight. This means it's good for strengthening your bones (the spine, pelvis and leg bones). All you need are comfortable walking shoes.
- **Gardening.** This is a way of enjoying some physical activity outdoors. It can also be therapeutic to watch your garden grow. If you don't have access to a garden, there are schemes across the UK where you can garden, grow fruit and vegetables or take part in nature conservation, for example green gyms. Visit your local authority's website to see what schemes might be available near you.

- **Running and jogging.** These can be considered as vigorous activity. They are great for your heart and lungs, and they are weight-bearing exercises. These activities are high impact and may put stress on your spine and joints. So if you have bone or joint problems, you should choose something gentler, such as jogging on a trampoline.
- **Cycling and swimming.** These are good for your heart and lungs. They strengthen your muscles but put very little strain on your joints, because they are weight-supported exercises. They can be good activities if you have bone or joint pain. Swimming is particularly beneficial if you have lymphoedema. Swimming is not recommended if you have irritated skin due to radiotherapy, or if you're having chemotherapy – especially if you have a PICC line or central line.
- **Group exercise.** There are other aerobic exercise options that you could do with a friend or a group. These include aerobics classes, dancing, golf, tennis, badminton and bowling.

Exercise intensity

If you haven't been active for a long time, increase your activity slowly. Try to do a little more in time and distance each week. When you're comfortable doing an activity for longer, you can think about increasing the intensity, for example walking the same distance but in a shorter time.

This is how it feels to be active at different intensities:

- **Light intensity** – You are breathing and talking easily and it doesn't feel like there's a lot of effort involved.
- **Moderate intensity** – Your breathing is quicker and deeper, but you are able to talk. Your body warms up, your face has a healthy glow and your heart is beating faster than normal but not racing.
- **Vigorous or high intensity** – You are breathing very hard, so you can't carry on a conversation. Your heartbeat feels fast.

Resistance/strength exercises

These exercises involve making your muscles work harder than usual, against some form of resistance. They strengthen muscles, bones and joints. They may also improve your balance. This is a good activity if you have, or are at risk of, bone problems. But it's not good if you have secondary bone cancer. Having good muscle strength makes it easier to do day-to-day things for yourself, so it can help you be more independent.

The exercises can be done with hand weights, machines or elastic bands. You can do simple exercises at home, such as lifting cans of food or bottles of water. There are more examples of the types of exercise you can do at home on the DVD in our guide **Move more: your guide to becoming more active**. You can order it by calling us on **0808 808 00 00**.

There are other simple resistance exercises you can do at home using a chair. These include 'sit to stand' and calf raises, or you can do press-ups against a wall. The NHS Choices website shows you how to do these and other simple exercises safely at home – visit **nhs.uk/tools/pages/exercises-for-older-people.aspx** These exercises are aimed at older people, but they are appropriate for people of any age who are not strong but want to start moving during treatment or while they are living with cancer.

Some exercise classes focus specifically on exercises you do while sitting down. Ask your GP or nurse if there are any in your local area, or call your local leisure centre to see what is available.

If you're doing a gym-based programme with resistance machines and free weights, make sure you get instruction from a qualified exercise specialist who has knowledge of cancer and its treatment.

Flexibility exercises

Having flexible joints helps you stay supple and prevent injuries and strains. Simple stretching exercises are a good way to start, especially if you have been unwell. The NHS Choices website has some stretches you can do – visit **nhs.uk/tools/pages/exercises-for-older-people.aspx** It's best to do these stretches as a daily routine. They will only take you a few minutes.

Yoga, tai chi and qi gong are also good for flexibility. They use breathing techniques combined with body movements. They can also help relax you and reduce stress.

Balance exercises

Yoga, tai chi, pilates, body balance and qi gong help increase balance and strength. Cycling and dancing are also good for your balance.

Later Life Training (see page 52) can send you booklets about simple balance exercises you can do at home. These are good for building strength, whatever your age.

The table on the next pages lists different activities and how they help improve your stamina, strength, flexibility and balance. It's been adapted from the British Heart Foundation's booklet **Get active, stay active**.

Activity	Aerobic/ stamina	Strength	Flexibility	Balance
Aerobic classes at a gym	✓	✓		✓
Badminton	✓		✓	✓
Brisk walking/ walking uphill	✓	✓		
Canoeing/ dragon boating (see IBCPC on page 52)	✓	✓	✓	
Climbing stairs	✓	✓		✓
Cycling	✓	✓		✓
Dancing	✓	✓	✓	✓
DIY		✓	✓	✓
Football	✓	✓		✓
Mowing the lawn	✓		✓	

Activity	Aerobic/ stamina	Strength	Flexibility	Balance
Pilates/ tai chi/ qi gong		✔	✔	✔
Vacuuming	✔		✔	
Washing the car	✔	✔		
Water aerobics	✔	✔		✔
Yoga		✔	✔	✔

If you're having treatment

Surgery

It's important to start moving around as soon as possible after surgery. This reduces the risk of complications such as blood clots. Depending on the operation, your surgeon, physiotherapist or nurse will tell you which activities you should avoid and for how long.

A physiotherapist or nurse may show you exercises to do when you get home. For example, women who have breast surgery need to do arm and shoulder exercises to improve their flexibility.

If you had surgery to your pelvis, you may be shown exercises to help strengthen your stomach (core) and pelvic floor muscles. Try to do these for as long as you were advised to. If you have pain, discomfort or swelling that stops you doing them, tell the physiotherapist or nurse.

Chemotherapy

Chemotherapy lowers the number of blood cells in your blood. If your number of white blood cells is low, you are more at risk of getting an infection. Your cancer doctor might advise you to avoid public places such as such as swimming pools or gyms until your white blood cells are back to a normal level.

We can send you more information about avoiding infection when you have reduced immunity. There is also a slideshow about avoiding infection at **macmillan.org.uk/avoidinginfection**

If you have a central line or PICC line, avoid swimming because of the risk of infection. You should also avoid vigorous upper body exercises, which could displace your line.

Platelets are cells that help the blood to clot. If your number of platelets is low, you are more at risk of bruising or bleeding. Your doctor may advise you to exercise gently until your platelets recover.

If your number of red blood cells is very low, you will feel very tired and sometimes breathless. This is called anaemia. If this happens, only do day-to-day activities until the anaemia improves.

Radiotherapy

If you have a skin reaction or redness due to radiotherapy, wear loose clothing when exercising to prevent rubbing against any areas of sensitive skin. Avoid swimming, as the chemicals in the water can irritate your skin. After treatment ends and any redness or skin reaction has gone, it's fine to swim again. Ask your radiotherapy team for advice about swimming during and after treatment.

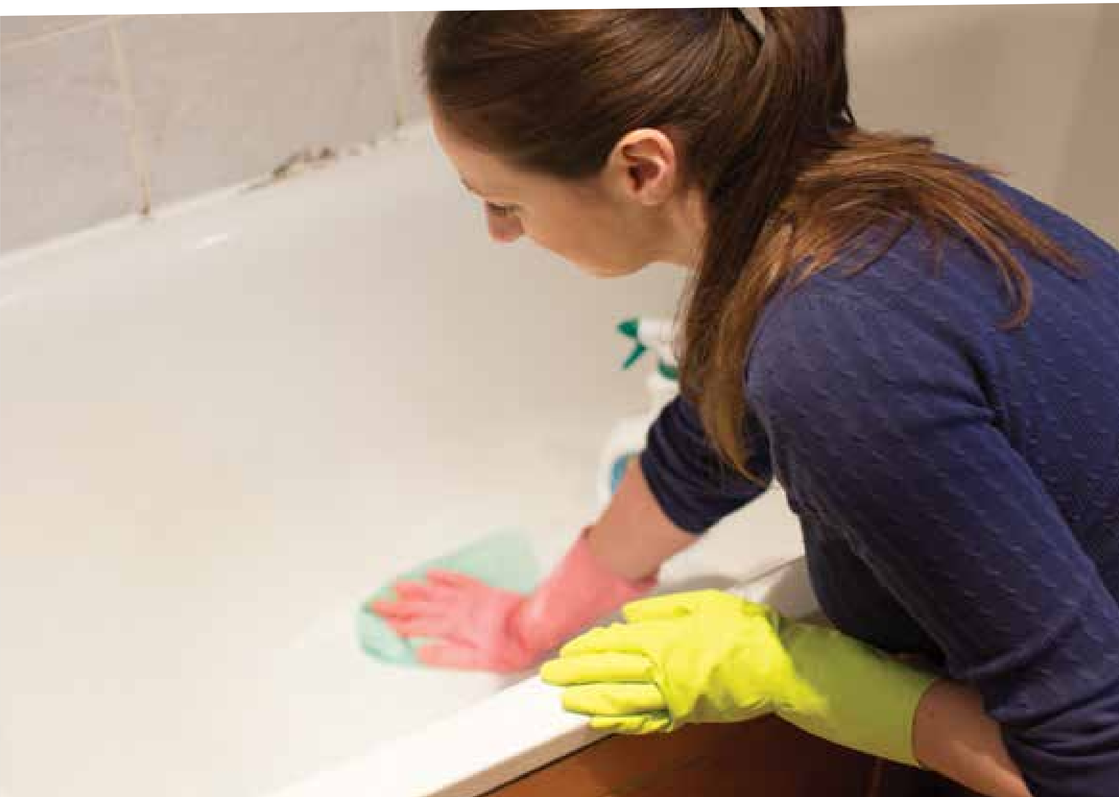

If you have side effects or other medical conditions

Some treatment side effects or other medical conditions can affect which physical activities are right for you.

Bone problems

If you have bone thinning or cancer in the bones, avoid high-impact activities. This is because there is more risk of you breaking (fracturing) a bone with these. High-impact activities are things that involve pounding or repetitive actions, for example jumping up and down on a hard surface or hitting a ball with a racket. They include running, jogging, football, tennis, squash, hockey and contact sports such as judo and karate. You should also avoid exercises where you repetitively bend forward at the waist, such as toe-touching and sit-ups.

It's safe for you to do low-impact activities. Good activities include:

- walking
- dancing
- climbing stairs
- swimming
- cycling
- light resistance exercises (see page 26).

It's also a good idea to do some exercises that improve your co-ordination and balance. This is to reduce your risk of falling. These exercises include dancing, body balance classes, tai chi and qi gong. Standing on one leg while holding on to a solid surface at home can help you improve your balance. You can make this more difficult by closing your eyes while balancing.

Peripheral neuropathy (nerve damage)

Some chemotherapy drugs can damage the nerves. This causes numbness or tingling in your hands or feet, muscle weakness or difficulty with balance and co-ordination.

If your feet or balance are affected, then running or brisk walking (especially on uneven surfaces) or walking up and down steps may not be the best activity for you. Cycling or swimming may be more suitable. Remember to check your feet regularly for cuts or blisters.

If you have numbness and tingling in your hands, it may be difficult or dangerous to use free weights or resistance bands.

Lymphoedema

Physical activity can help you reduce the risk of developing lymphoedema. Or if you have lymphoedema, physical activity can help you manage it.

If you have lymphoedema, the following may help:

- Always wear a compression garment when you exercise.
- Avoid doing lots of repetitive action with the affected limb.
- Strength exercises are safe for people with lymphoedema, and they may help prevent symptoms.
- Swimming can be helpful if you have lymphoedema, as it gently massages the lymphatic vessels. Ask your lymphoedema specialist for advice.
- Build up the physical activity involving the affected arm or leg slowly.

We have more information about lymphoedema. To order it, visit **be.macmillan.org.uk** or call us on **0808 808 00 00**.

Heart or lung problems

Most people with heart or lung problems can benefit from regular physical activity. Check with your doctor or specialist nurse before you start any exercise programme.

We have more information about heart health. To order it, visit **be.macmillan.org.uk** or call us on **0808 808 00 00**.

Medicines to thin the blood

If you're taking medicine to thin the blood, you will bruise more easily. Avoid high-impact activities (see page 32), as you could get knocked or fall over. Take extra care during activities to avoid damaging your skin.

Stoma

If you have had surgery for bowel cancer, you may have a stoma. You will need to learn new skills to manage this. You may also need time to adjust to the changes in your body. This may affect what physical activity you choose to do. Your specialist nurse, sometimes called a stoma care nurse, will be able to help you think about how you can start doing some physical activity with the stoma in place.

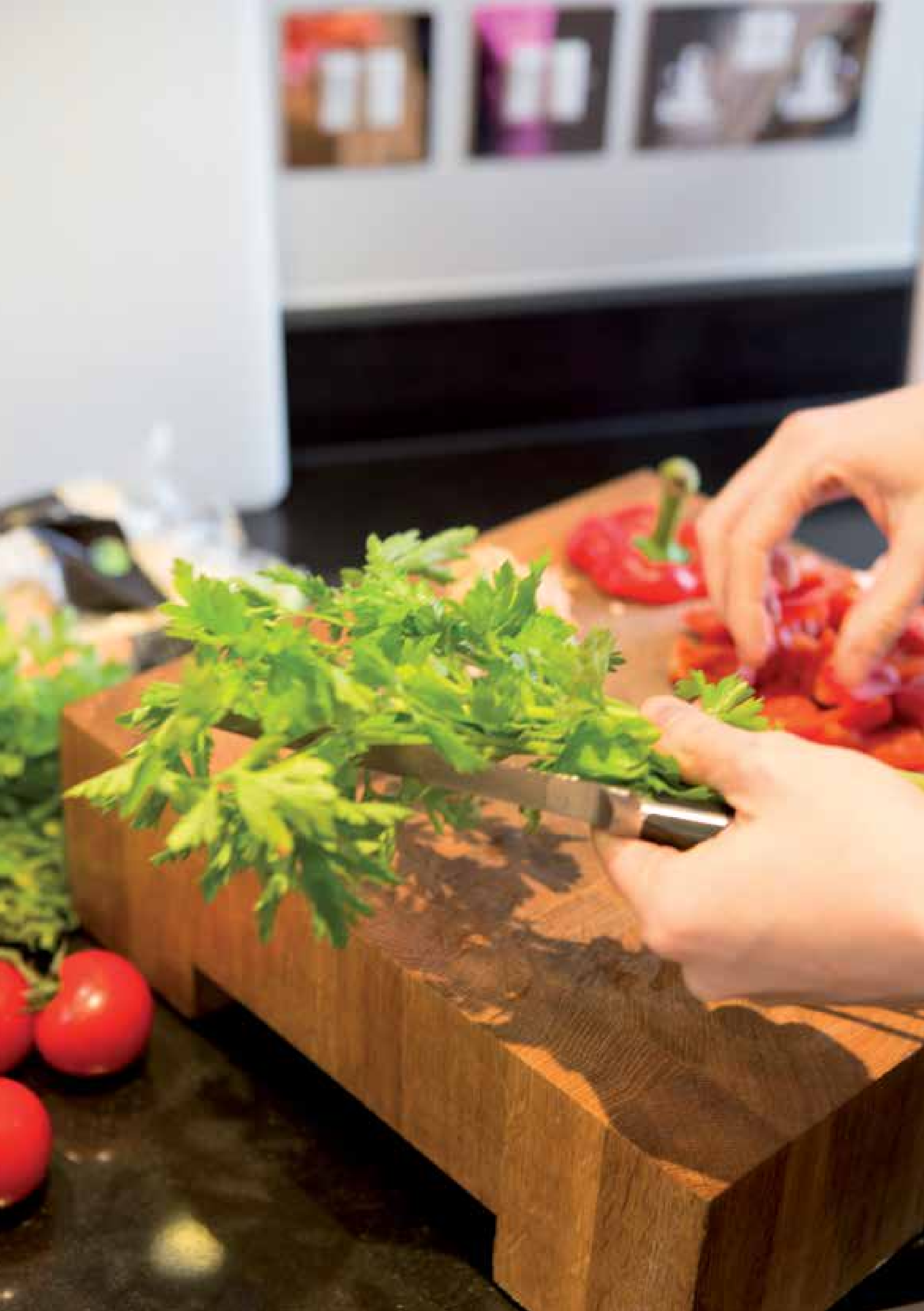

Who can help?

If you haven't been active before or for a long time, or if you feel nervous about starting physical activity, it can help to get advice. On the next pages are people and organisations that can help you.

Your doctor or nurse

It's best to talk to your cancer doctor, specialist nurse or GP before you start exercising. They can give you advice on whether it is safe for you to exercise and any precautions you need to take. Some GPs, cancer treatment centres and support groups have exercise referral schemes – see page 38. These referral programmes support people to change their behaviours and get healthier and more active after illnesses such as cancer. There may be an exercise programme that's part of some research at your cancer treatment centre. Ask your cancer doctor or nurse for more information.

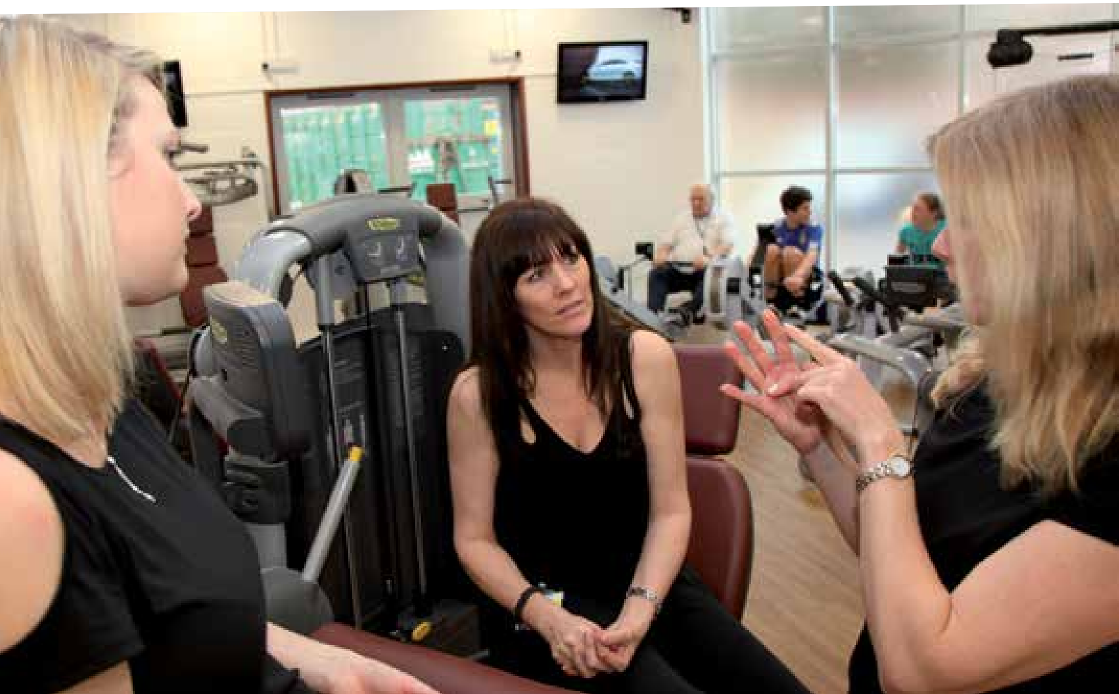

A physiotherapist or cancer exercise specialist

Your GP or cancer specialist can refer you to a physiotherapist or a qualified cancer exercise specialist if you need one. You may also be able to refer yourself to a physiotherapist. They can help you:

- decide on an individual exercise programme that is safe and effective
- become an independent exerciser
- increase your physical fitness
- improve your energy, strength, flexibility, co-ordination and balance
- manage the side effects or after-effects of treatment
- enjoy and increase your physical activity
- avoid injuries.

You can ask them questions such as:

- 'I've never exercised before – how should I start?'
- 'How can I improve my shortness of breath and feel less tired?'
- 'How can I improve my balance?'
- 'Can I improve the strength of my hands, arms or legs?'
- 'Which exercises can help me get up the stairs?'

If you have more specific needs, they may be able to arrange a rehabilitation programme for you.

Exercise referral schemes

Many people find that exercising in a group keeps them more motivated. If you think this might help, or you feel you need close supervision from an expert, you may want to join an exercise referral scheme. They are mostly held at local community centres, healthy living centres, leisure centres and libraries. Ask your GP or cancer doctor about any in your area. Not all areas have an exercise referral scheme, or the schemes may be restricted to certain medical conditions.

Specially trained exercise professionals manage many of these schemes. But it's a good idea to check whether they have been trained to work with people who have or have had cancer.

When they make your referral, your doctor will share some information about your health with the exercise professional. They will keep this confidential.

Your trainer will explain the benefits and risks of increasing your physical activity. You'll be asked to give your consent. This means you agree to the exercise plan and understand the benefits and risks. They will advise you on whether it's best to exercise in a group or on your own. Your trainer will match the activities to your individual needs and ability.

Some areas also have schemes in care homes, hospices and day centres. They often include activities such as chair-based exercises, walking, dancing, pilates, gentle exercise to music, circuits, tai chi and yoga.

An occupational therapist

Your GP or cancer doctor may refer you to an occupational therapist. They can advise you on how to pace yourself and manage tiredness.

They can also suggest changes to your home that will make it safer and help you be more independent. Doing simple things for yourself in the home is a way of increasing your physical activity. If things are easier at home, you will also have more time and energy to do activities such as walking or gardening.

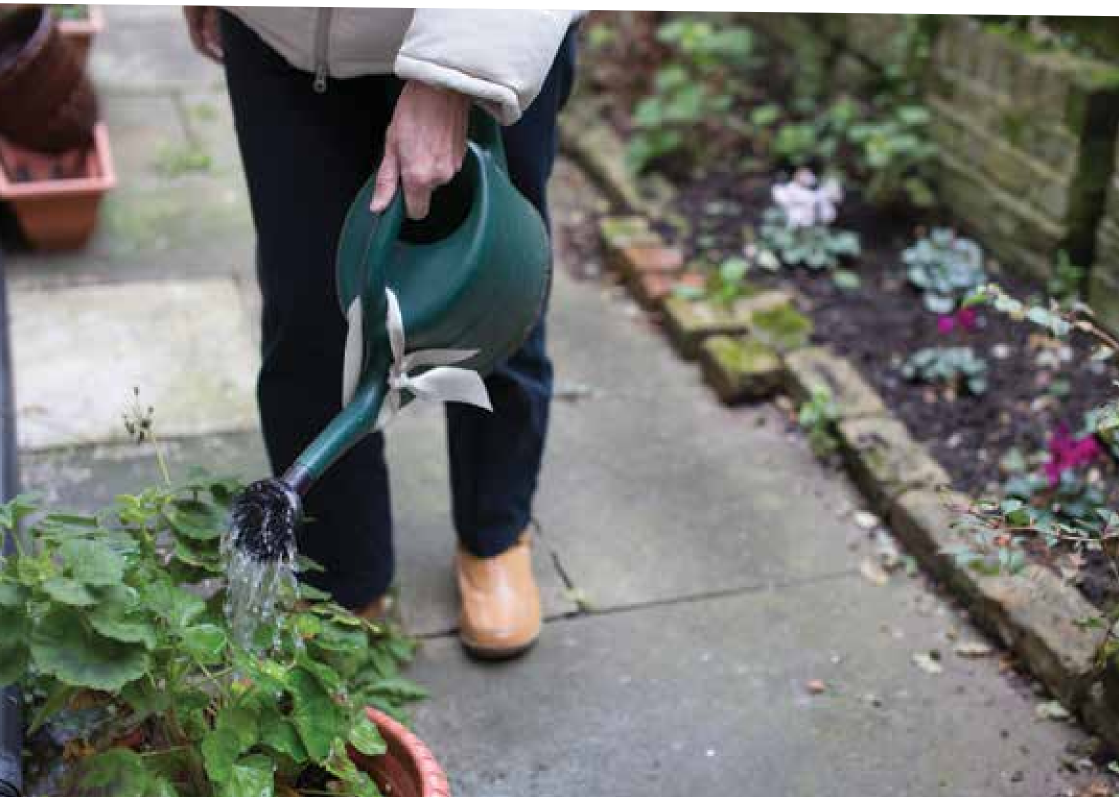

Getting started

Our guide **Move more: your guide to becoming more active** can help you get started. It includes goal-setting pages, tips and advice. It also includes a DVD, which shows you aerobic, strength and flexibility exercises, at a variety of intensity levels. To order our **Move more** guide or the DVD, visit **be.macmillan.org.uk**

When you start getting more active, begin by doing something you enjoy and that fits in with your life. This could be a brisk walk with friends, playing with your children or grandchildren in the park, gardening or walking to the shops.

Here are some more ideas:

- See if your family or friends want to join you to do some activity.
- Join a walking group and make friends while you walk – see page 43.
- Walk or cycle to the shops, to see friends or to work.
- Try swimming, cycling, dancing or gardening.
- Play a sport, such as badminton, golf, table tennis or bowls.
- Try a new walking sport, such as walking football or walking netball.
- Try stretching exercises, such as yoga, tai chi or pilates.
- Visit your local leisure centre (or its website) to see what activities are available.
- Ask your GP to refer you to a structured exercise referral scheme or a physiotherapist.

It's not always easy to get active for the first time, or to return to activity during or after cancer. Having clear goals, staying motivated and having support can all be very helpful. If your family and friends can take part with you, that can help you meet your goals. Our **Move more** guide can also help you achieve this.

Here are some things that other people have found helpful:

- Remind yourself of the benefits and reasons why you're doing this.
- Set yourself short-term (one-week) and long-term (six-month) goals that you can feel you can achieve at your own pace. This might be getting up from the sofa regularly, going for a walk or doing an exercise class. It might help motivate you if you have a long-term goal to aim for, such a sponsored local event.
- Slowly increase the amount you do. If you have a setback due to bad weather, illness or a holiday break, return to an easier stage of your activity plan.
- Keep a record of how active you've been and how you feel, so you can see your progress. There's space to do this in our **Move more** guide.
- Share your plans with other people who are supportive. They might join you, help with commitments like childminding, or even drive you to the venue.
- Try being active with other people such as family or friends, or join a group or club.
- Make sure the activities are fun and enjoyable. Some people find it's helpful to do a variety of activities.
- Don't be put off if you don't achieve a goal. Think about why you weren't able to achieve it and plan a new one. Sometimes it can take longer to develop fitness after treatment, so you may need to change the dates you expect to achieve your goals.

What activities are available near you?

There are lots of ways you can find out about activities in your area:

- Contact your local council. Look on the website or call to find out what activities are provided in parks and leisure centres.
- Ask your GP if they can refer you to any specialist services. This might be an exercise referral scheme, falls prevention (if you're worried about falling) or physiotherapy (for example if you've had surgery and have problems with range of movement).
- Use online search tools:
    - Search for Move More events in your local area on our website. Visit **macmillan.org.uk/inyourarea**
    - On the Be Inspired website, you can search for sport and fitness venues, clubs and activities across the UK. You can search by activity or location. Visit **beinspireduk.org**
    - There is a national search tool for England on the NHS Choices website. Visit **nhs.uk/livewell/fitness/pages/whybeactive.aspx** and enter your postcode. NHS Choices also has suggested exercise videos and podcasts.
    - There is a national search tool for Scotland at **activescotland.org.uk** Enter your postcode or town to find activities near you.
    - In Wales, you can use the website **sportwales.org.uk** to search for activities near you.
    - In Northern Ireland, you can use the website **sportni.net** to search for activities near you.

Walking groups

Walking groups are an enjoyable, social way to become active. There are free, guided health walks across the UK. You can search for a walk near you using the details below.

Let's Walk Cymru (Wales)

www.letswalkcymru.org.uk
Tel 029 2064 4308

Paths for All (Scotland)

www.pathsforall.org.uk
Tel 0125 921 8888

Ramblers

www.ramblers.org.uk
Tel 020 7339 8500 (England)
0131 472 7006 (Scotland)
029 2064 4308 (Wales)

Walking for Health (England)

www.walkingforhealth.org.uk
Tel 020 7339 8541

Walk NI (Northern Ireland)

www.walkni.com
Tel 028 9030 3930

'I found the Walking for Health leaflet in my library. It was one of the best things I've ever done. I feel like I've found the real me again. I am fitter, happier and more confident.'

Jermaine

Sports

National sports councils can give you contact details for individual sports' governing bodies. They can also help you find facilities near you. These are the UK's sports councils:

Sport England
www.sportengland.org
Tel 020 7273 1551

Sport Scotland
www.sportscotland.org.uk
Tel 0141 534 6500

Sport Wales
www.sportwales.org.uk
Tel 0300 300 3111

Sport Northern Ireland
www.sportni.net
Tel 028 9038 1222

'I have found that to make sure I do exercise, I have to plan it in advance. Otherwise it won't happen.'

Beverly

Mobility and disability organisations

There are specific organisations that can help if you have mobility problems or a disability:

Disability Sports Northern Ireland
www.dsni.co.uk

Disability Sport Wales
www.disabilitysportwales.com

English Federation of Disability Sport (EFDS)
www.efds.co.uk

Scottish Disability Sport
www.scottishdisabilitysport.com

WheelPower
www.wheelpower.org.uk

We have listed more organisations on pages 52–55 that can help you with physical activity.

Next steps

Once you've started, you will probably find that being active becomes an enjoyable part of the way you live. Many people notice the benefits quite quickly. You'll feel less tired and stressed, and have more confidence. Noticing these changes and knowing the benefits to your health can keep you motivated, even on days when it's difficult. If you're struggling, don't be hard on yourself. Just remind yourself of all the benefits.

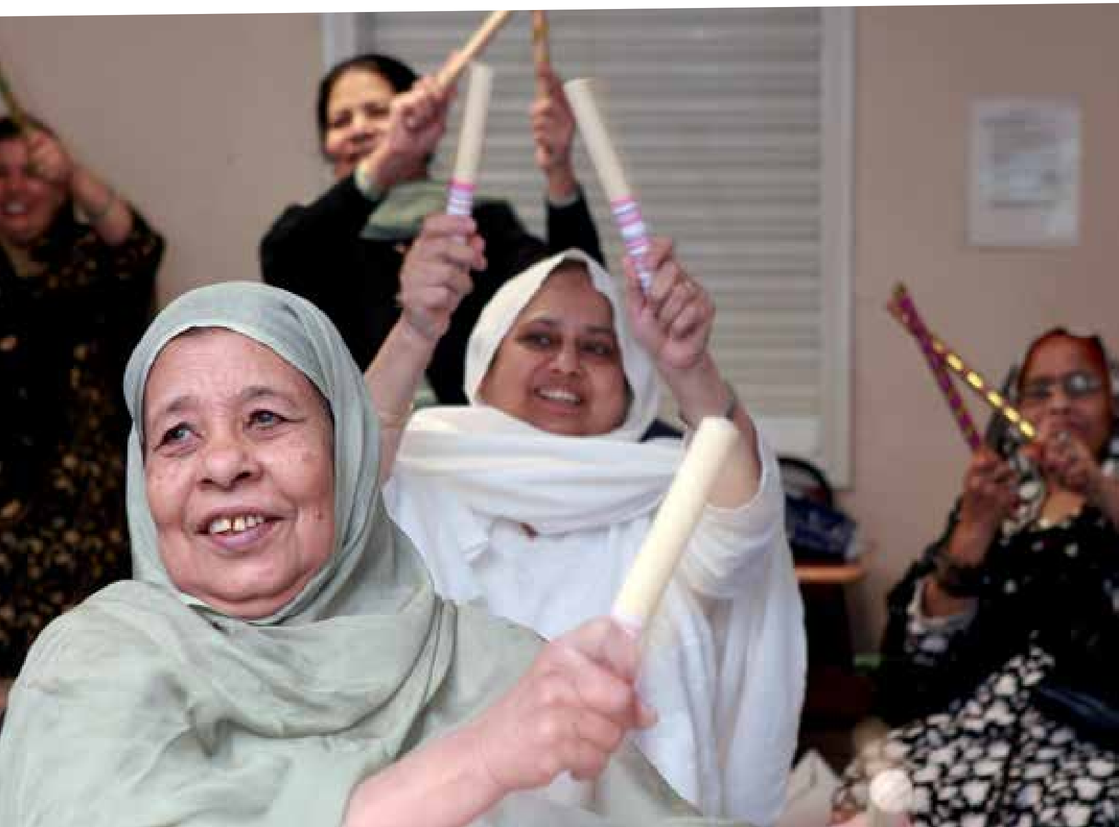

About our information

We provide expert, up-to-date information about cancer. And all our information is free for everyone.

Order what you need

You may want to order more leaflets or booklets like this one. Visit **be.macmillan.org.uk** or call us on **0808 808 00 00**.

We have booklets on different cancer types, treatments and side effects. We also have information about work, financial issues, diet, life after cancer and information for carers, family and friends.

All of our information is also available online at **macmillan.org.uk/cancerinformation** There you'll also find videos featuring real-life stories from people affected by cancer, and information from health and social care professionals.

Other formats

We also provide information in different languages and formats, including:

- audiobooks
- Braille
- British Sign Language
- Easy Read booklets
- ebooks
- large print
- translations.

Find out more at **macmillan.org.uk/otherformats** If you'd like us to produce information in a different format for you, email us at **cancerinformationteam@macmillan.org.uk** or call us on **0808 808 00 00**.

Help us improve our information

We know that the people who use our information are the real experts. That's why we always involve them in our work. If you've been affected by cancer, you can help us improve our information.

We give you the chance to comment on a variety of information including booklets, leaflets and fact sheets.

If you'd like to hear more about becoming a reviewer, email **reviewing@macmillan.org.uk** You can get involved from home whenever you like, and we don't ask for any special skills – just an interest in our cancer information.

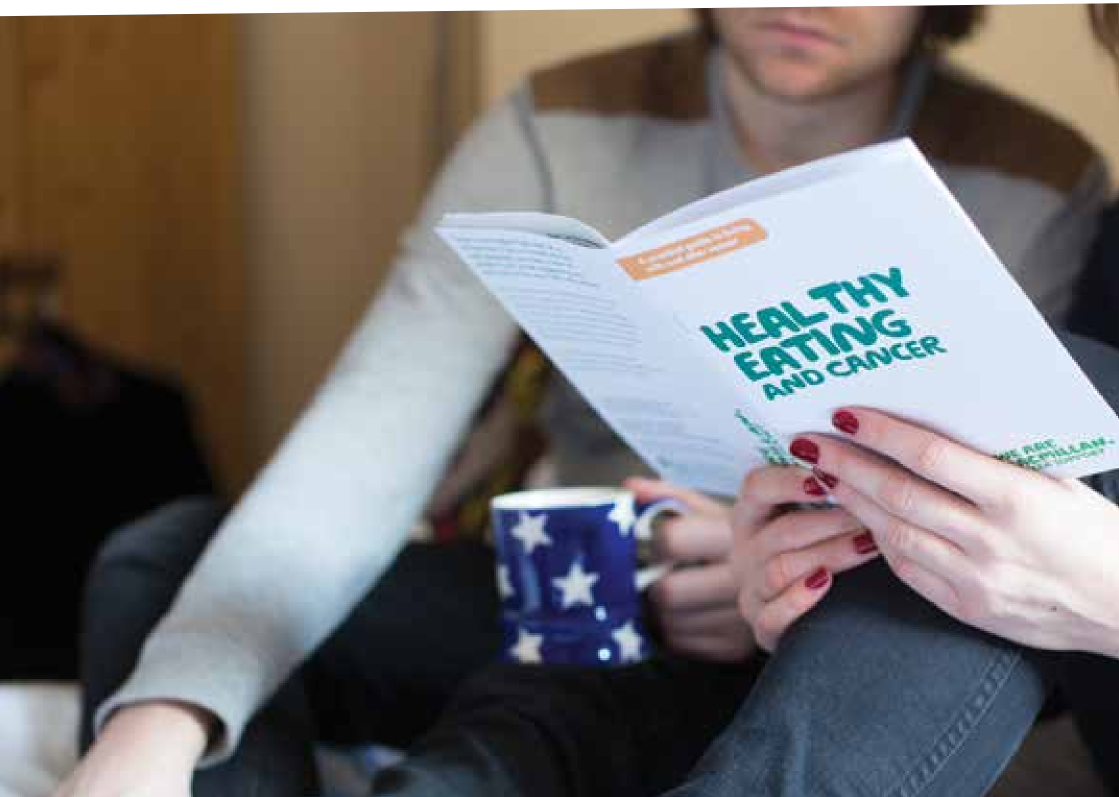

Other ways we can help you

At Macmillan, we know how a cancer diagnosis can affect everything, and we're here to support you. No one should face cancer alone.

Talk to us

If you or someone you know is affected by cancer, talking about how you feel and sharing your concerns can really help.

Macmillan Support Line

Our free, confidential phone line is open Monday–Friday, 9am–8pm. Our cancer support specialists can:

- help with any medical questions you have about your cancer or treatment
- help you access benefits and give you financial advice
- be there to listen if you need someone to talk to
- tell you about services that can help you in your area.

Call us on **0808 808 00 00** or email us via our website, **macmillan.org.uk/talktous**

Information centres

Our information and support centres are based in hospitals, libraries and mobile centres. There, you can speak with someone face to face.

Visit one to get the information you need, or if you'd like a private chat, most centres have a room where you can speak with someone alone and in confidence.

Find your nearest centre at **macmillan.org.uk/informationcentres** or call us on **0808 808 00 00**.

Talk to others

No one knows more about the impact cancer can have on your life than those who have been through it themselves. That's why we help to bring people together in their communities and online.

Support groups

Whether you are someone living with cancer or a carer, we can help you find support in your local area, so you can speak face to face with people who understand. Find out about support groups in your area by calling us or by visiting **macmillan.org.uk/selfhelpandsupport**

Online community

Thousands of people use our online community to make friends, blog about their experiences and join groups to meet other people going through the same things. You can access it any time of day or night. Share your experiences, ask questions, or just read through people's posts at **macmillan.org.uk/community**

The Macmillan healthcare team

Our nurses, doctors and other health and social care professionals give expert care and support to individuals and their families. Call us or ask your GP, consultant, district nurse or hospital ward sister if there are any Macmillan professionals near you.

'Everyone is so supportive on the online community, they know exactly what you're going through. It can be fun too. It's not all just chats about cancer.'

Mal

Help with money worries

Having cancer can bring extra costs such as hospital parking, travel fares and higher heating bills. If you've been affected in this way, we can help.

Financial guidance

Our financial guidance team can give you advice on mortgages, pensions, insurance, borrowing and savings.

Help accessing benefits

Our benefits advisers can offer advice and information on benefits, tax credits, grants and loans. They can help you work out what financial help you could be entitled to. They can also help you complete your forms and apply for benefits.

Macmillan Grants

Macmillan offers one-off payments to people with cancer. A grant can be for anything from heating bills or extra clothing to a much-needed break.

Call us on **0808 808 00 00** to speak to a financial guide or benefits adviser, or to find out more about Macmillan Grants. We can also tell you about benefits advisers in your area. Visit **macmillan.org.uk/financialsupport** to find out more about how we can help you with your finances.

Help with work and cancer

Whether you're an employee, a carer, an employer or are self-employed, we can provide support and information to help you manage cancer at work. Visit **macmillan.org.uk/work**

Macmillan's My Organiser app

This free mobile app can help you manage your treatment, from appointment times and contact details, to reminders for when to take your medication. Search 'My Organiser' on the Apple App Store or Google Play on your phone.

Other useful organisations

There are lots of other organisations that can give you information or support.

Organisations that can help with physical activity

Cancer Rehabilitation
Email info@canrehab.co.uk
www.canrehab.co.uk
Runs training workshops for health and fitness professionals on developing and providing safe and effective exercise-based cancer rehabilitation programmes.

Cyclists Fighting Cancer
Eversfield,
Shipston Road,
Alderminston CV37 8NY
Tel 0300 500 4040
Email info@cyclistsfc.org.uk
www.cyclistsfc.org.uk
Provides bikes, adapted tricycles and equipment to children and young people whose lives have been affected by cancer.

International Breast Cancer Paddlers' Commission (IBCPC)
Email committee@ibcpc.com
www.ibcpc.com
Can give details of UK dragon boating groups. There is an international movement encouraging breast cancer survivors to improve their physical health and social wellbeing through dragon boating.

Later Life Training
Silver Cottage,
Main Street,
Killin FK21 8UT
Tel 01838 300 310
Email
info@laterlifetraining.co.uk
www.laterlifetraining.co.uk
Has useful leaflets about simple exercises you can do at home, which can improve your strength and balance whatever your age.

National Osteoporosis Society

Camerton,
Bath BA2 0PJ
Tel 0808 800 0035
(Mon, Wed–Fri, 9am–5pm,
Tue, 11am–7pm)
Email nurses@nos.org.uk
www.nos.org.uk
UK charity dedicated to improving the diagnosis, prevention and treatment of osteoporosis.

General cancer support organisations

Cancer Black Care

79 Acton Lane,
London NW10 8UT
Tel 020 8961 4151
Email
info@cancerblackcare.org.uk
www.cancerblackcare.org.uk
Offers information and support for people with cancer from ethnic communities, their friends, carers and families.

Cancer Focus Northern Ireland

40–44 Eglantine Avenue,
Belfast BT9 6DX
Tel 0800 783 3339
(Mon–Fri, 9am–1pm)
Email hello@cancerfocusni.org
www.cancerfocusni.org
Offers a variety of services to people affected by cancer, including a free helpline, counselling and links to local support groups.

Cancer Support Scotland

Calman Cancer
Support Centre,
75 Shelley Road,
Glasgow G12 0ZE
Tel 0800 652 4531
Email info@cancersupport scotland.org
www.cancersupport scotland.org
Runs cancer support groups throughout Scotland. Also offers free complementary therapies and counselling to anyone affected by cancer.

Health and Social Care in Northern Ireland

www.hscni.net
Provides information about health and social care services in Northern Ireland.

Healthtalk

www.healthtalk.org
www.youthhealthtalk.org
Contains information about some cancers and has video and audio clips of people talking about their experiences of cancer and its treatments.

Irish Cancer Society

43–45 Northumberland Road, Dublin 4
Tel 1800 200 700
(Mon–Thu, 9am–7pm, Fri, 9am–5pm)
Email helpline@irishcancer.ie
www.cancer.ie
National cancer charity offering information, support and care to people affected by cancer. Has a helpline staffed by specialist cancer nurses. You can also chat to a nurse online and use the site's message board.

Maggie's Centres

Tel 0300 123 1801
Email enquiries@maggiescentres.org
www.maggiescentres.org
Provide information about cancer, benefits advice, and emotional or psychological support. Find details for your local centre on the website.

NHS Choices

www.nhs.uk
The UK's biggest health information website. Also has service information for England.

NHS Direct Wales

www.nhsdirect.wales.nhs.uk
NHS health information site for Wales.

NHS Inform

www.nhsinform.co.uk
NHS health information site for Scotland.

Patient UK

www.patient.co.uk
Provides people in the UK with good-quality information about health and disease. Includes evidence-based information leaflets on a wide variety of medical and health topics. Also reviews and links to many health and illness-related websites.

Tenovus

Head Office,
Gleider House,
Ty Glas Road,
Cardiff CF14 5BD
Tel 0808 808 1010
(Mon–Sun, 8am–8pm)
www.tenovus.org.uk
Aims to help everyone get equal access to cancer treatment and support. Funds research and provides mobile cancer support units, a free helpline, an 'Ask the nurse' service on the website and benefits advice.

You can search for more organisations on our website at **macmillan.org.uk/organisations** or call us on **0808 808 00 00**.

YOUR NOTES AND QUESTIONS

Disclaimer

We make every effort to ensure that the information we provide is accurate and up to date but it should not be relied upon as a substitute for specialist professional advice tailored to your situation. So far as is permitted by law, Macmillan does not accept liability in relation to the use of any information contained in this publication, or third-party information or websites included or referred to in it. Some photographs are of models.

Thanks

This booklet has been written, revised and edited by Macmillan Cancer Support's Cancer Information Development team. It has been approved by our senior reviewer, Dr Anna Campbell, Associate Professor at Edinburgh Napier University and Director of CanRehab, and by our Chief Medical Editor, Dr Tim Iveson, Macmillan Consultant Medical Oncologist.

With thanks to: Jo Foster, Physical Activity Programme Lead; Maggi Hardcastle, Clinical Specialist Physiotherapist; Siobhan Harkin, Macmillan Specialist Physiotherapist; Kate Jones, Clinical Specialist Physiotherapist; Claire Lait, Head and Neck Cancer Specialist Physiotherapist; Lorna Malcolm, Physiotherapist; Katharine Malhotra, Lecturer Practitioner; Tamzin Marsh, Marketing Officer; Professor Alan Maryon-Davis, Faculty of Public Health; Aileen McCartney, Specialist Palliative Care Physiotherapist; John Saxton, Professor of Clinical Exercise Physiology; Clare Stevinson, Lecturer in Physical Activity and Health; Professor Rob Thomas, Macmillan Consultant Oncologist; and Justin Webb, Physical Activity Engagement Manager. Thanks also to the people affected by cancer who reviewed this edition, and those who shared their stories.

Sources

We've listed a sample of the sources used in this publication below. If you'd like further information about the sources we use, please contact us at **bookletfeedback@macmillan.org.uk**

Feuerstein M. Handbook of cancer survivorship. Springer. 2007.
Macmillan Cancer Support. The importance of physical activity for people living with and beyond cancer: a concise evidence review. 2015.
Schmitz K, Courneya K, Matthews C, et al. American College of Sports Medicine roundtable on exercise guidelines for cancer survivors. Medicine and science in sports and exercise. 2010. 42.
Thomas RJ et al. Physical activity after cancer: an evidence review of international literature. British journal of medical practitioners. 2014. 7(1) 708.

The infographic on page 21 is based on data from **gov.uk**

Can you do something to help?

We hope this booklet has been useful to you. It's just one of our many publications that are available free to anyone affected by cancer. They're produced by our cancer information specialists who, along with our nurses, benefits advisers, campaigners and volunteers, are part of the Macmillan team. When people are facing the toughest fight of their lives, we're there to support them every step of the way.

We want to make sure no one has to go through cancer alone, so we need more people to help us. When the time is right for you, here are some ways in which you can become a part of our team.

5 WAYS YOU CAN HELP SOMEONE WITH CANCER

Share your cancer experience
Support people living with cancer by telling your story, online, in the media or face to face.

Campaign for change
We need your help to make sure everyone gets the right support. Take an action, big or small, for better cancer care.

Help someone in your community
A lift to an appointment. Help with the shopping. Or just a cup of tea and a chat. Could you lend a hand?

Raise money
Whatever you like doing you can raise money to help. Take part in one of our events or create your own.

Give money
Big or small, every penny helps. To make a one-off donation see over.

Call us to find out more

0300 1000 200

macmillan.org.uk/getinvolved

Please fill in your personal details

Mr/Mrs/Miss/Other

Name

Surname

Address

Postcode

Phone

Email

Please accept my gift of £

(Please delete as appropriate)
I enclose a cheque / postal order / Charity Voucher made payable to Macmillan Cancer Support

OR debit my:
Visa / MasterCard / CAF Charity Card / Switch / Maestro

Card number

Valid from

Expiry date

Issue no

Security number

Signature

Date / /

Don't let the taxman keep your money

Do you pay tax? If so, your gift will be worth 25% more to us – at no extra cost to you. All you have to do is tick the box below, and the tax office will give 25p for every pound you give.

☐ I am a UK tax payer and I would like Macmillan Cancer Support to treat all donations I make or have made to Macmillan Cancer Support in the last 4 years as Gift Aid donations, until I notify you otherwise.

I understand that if I pay less Income Tax and/or Capital Gains Tax than the amount of Gift Aid claimed on all my donations in that tax year it is my responsibility to pay any difference. I understand Macmillan Cancer Support will reclaim 25p of tax on every £1 that I give.

Macmillan Cancer Support and our trading companies would like to hold your details in order to contact you about our fundraising, campaigning and services for people affected by cancer. If you would prefer us not to use your details in this way please tick this box. ☐

In order to carry out our work we may need to pass your details to agents or partners who act on our behalf.

FRSB give with confidence

If you'd rather donate online go to macmillan.org.uk/donate

Please cut out this form and return it in an envelope (no stamp required) to:
Supporter Donations, Macmillan Cancer Support, FREEPOST LON15851, 89 Albert Embankment, London SE1 7UQ

27530

More than one in three of us will get cancer. For most of us it will be the toughest fight we ever face. And the feelings of isolation and loneliness that so many people experience make it even harder. But you don't have to go through it alone. The Macmillan team is with you every step of the way.

We are the nurses and therapists helping you through treatment. The experts on the end of the phone. The advisers telling you which benefits you're entitled to. The volunteers giving you a hand with the everyday things. The campaigners improving cancer care. The community there for you online, any time. The supporters who make it all possible.

Together, we are all Macmillan Cancer Support.

For cancer support every step of the way, call Macmillan on 0808 808 00 00 (Mon–Fri, 9am–8pm) or visit macmillan.org.uk

Hard of hearing? Use textphone 0808 808 0121, or Text Relay. Non-English speaker? Interpreters available. Braille and large print versions on request.

© Macmillan Cancer Support, June 2016. 4th edition. MAC12515. Next planned review 2018. Macmillan Cancer Support, registered charity in England and Wales (261017), Scotland (SC039907) and the Isle of Man (604). Printed using sustainable material. Please recycle.

Health & care information you can trust

The Information Standard Certified Member

WE ARE MACMILLAN. CANCER SUPPORT
