# Supplementary material for: The Effect of the Move More Pack on the Physical Activity of Cancer Survivors: Protocol for a Randomized Waiting List Control Trial with Process Evaluation
Source: JMIR Res Protoc. 2017 Nov 9;6(11):e220. doi: 10.2196/resprot.7755 (PMC5701086; doi:10.2196/resprot.7755)
Supplement: Multimedia Appendix 3 [file resprot_v6i11e220_app3.pdf]

RECORD YOUR DAILY ACHIEVEMENTS

WE ARE MACMILLAN. CANCER SUPPORT

Keep this somewhere you can see it easily, such as on your fridge, and record how you're doing each day.

'Physical activity lifts your spirits and can be enjoyed by everyone. Do something you enjoy: walking, gardening, swimming – the list is endless. Not only will you feel healthier, but you may meet new friends along the way.'

Rachel Bryce, Breast Care Specialist Nurse, Bedford Hospital

'Taking small steps every day over a period of time to become more physically active has given me my life back.'

Lorraine, diagnosed with lung cancer in 2007

	Monday	Tuesday	Wednesday	Thursday	Friday	Saturday	Sunday
Week starting / /	week one						
Week starting / /	week two						
Week starting / /	week three						
Week starting / /	week four						

If you have questions, need support or just want to chat, you can call the Macmillan Support Line free on **0808 808 00 00** (Monday–Friday, 9am–8pm).

Turn over for weeks 5–12

Record your daily achievements

	Monday	Tuesday	Wednesday	Thursday	Friday	Saturday	Sunday
Week starting / /	week five						
Week starting / /	week six						
Week starting / /	week seven						
Week starting / /	week eight						

	Monday	Tuesday	Wednesday	Thursday	Friday	Saturday	Sunday
Week starting / /	week nine						
Week starting / /	week ten						
Week starting / /	week eleven						
Week starting / /	week twelve						

For more information and support as you get active, visit **macmillan.org.uk/beactive**

© Macmillan Cancer Support, July 2016. 1st edition. MAC16044_chart. Next planned review 2019. Macmillan Cancer Support, registered charity in England and Wales (261017), Scotland (SC039907) and the Isle of Man (604).
Printed using sustainable material. Please recycle.
