# Supplementary figures and images for: The Effect of the Move More Pack on the Physical Activity of Cancer Survivors: Protocol for a Randomized Waiting List Control Trial with Process Evaluation
Source: JMIR Res Protoc. 2017 Nov 9;6(11):e220. doi: 10.2196/resprot.7755 (PMC5701086; doi:10.2196/resprot.7755)

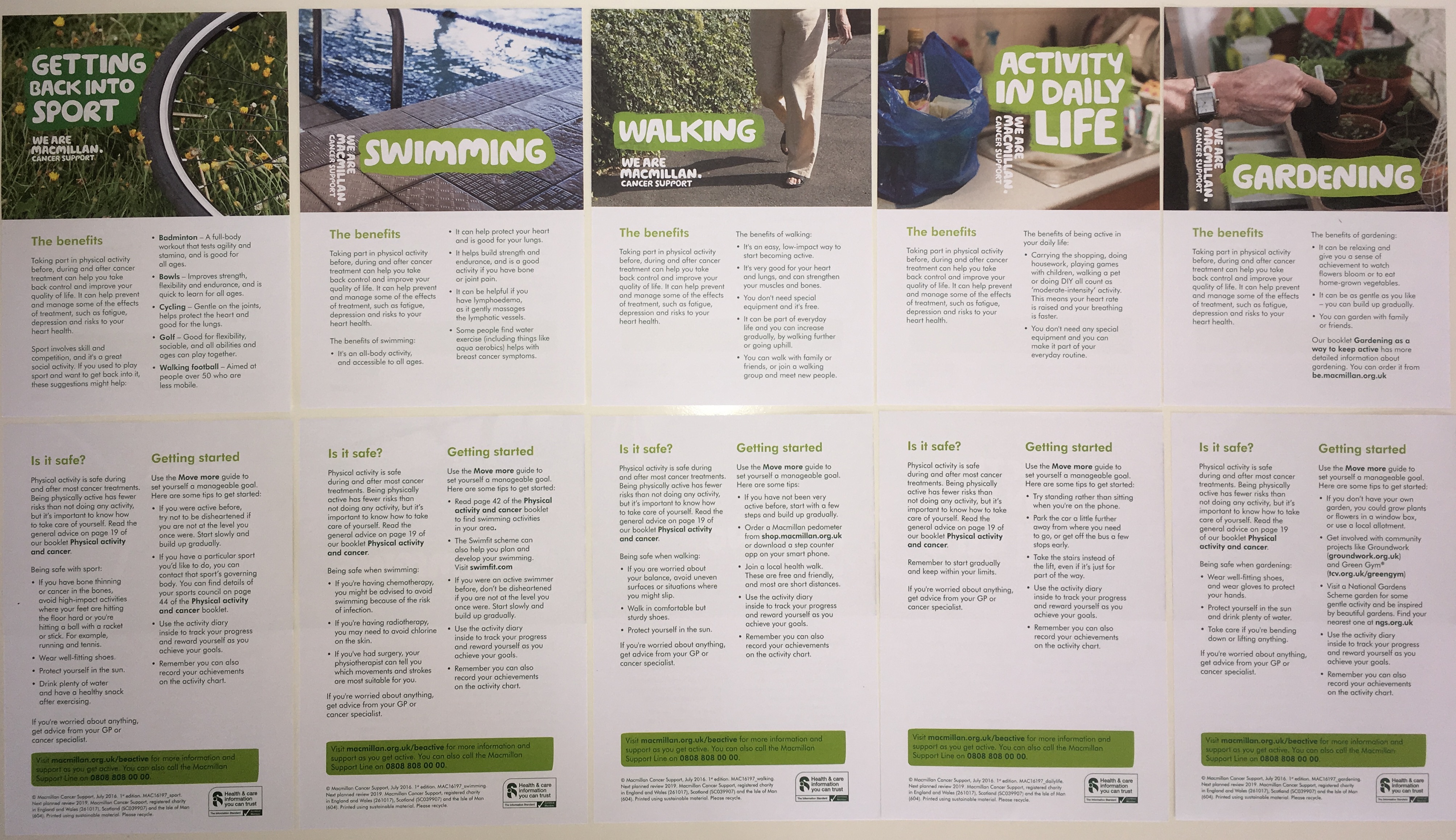

Supplement: Multimedia Appendix 4 [file resprot_v6i11e220_app4.JPG]
