# Supplementary material for: The Effect of the Move More Pack on the Physical Activity of Cancer Survivors: Protocol for a Randomized Waiting List Control Trial with Process Evaluation
Source: JMIR Res Protoc. 2017 Nov 9;6(11):e220. doi: 10.2196/resprot.7755 (PMC5701086; doi:10.2196/resprot.7755)
Supplement: Multimedia Appendix 5 [file resprot_v6i11e220_app5.pdf]

Dear Insert First Name

Read on to find out more about the benefits of being active following a cancer diagnosis, including some examples from people who have felt the benefits of moving more, and some tips that you might find useful.

Remember to look out for another update next month.

The Physical Activity Team at Macmillan

Expert Advice

Watch this short video with advice from Consultant Oncologist, Professor Robert Thomas who discusses the benefits of physical activity during and after cancer treatment or read the brief summary below.

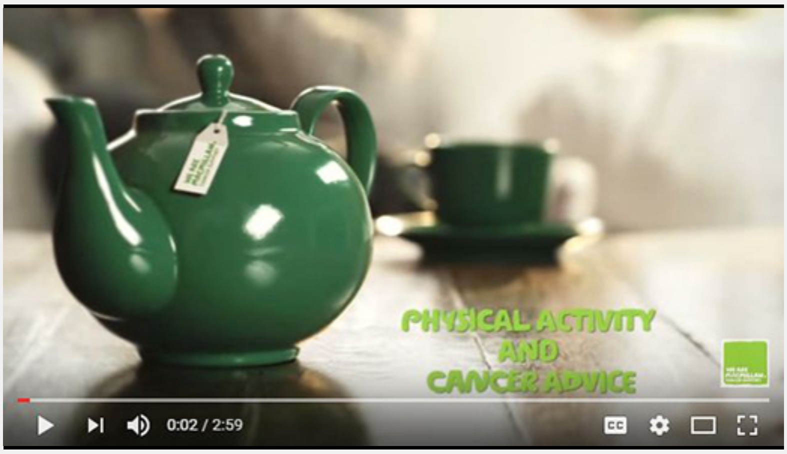

Physical activity helps patients in a number of ways:

- it can reduce some of the side effects of treatment, such as fatigue
- it makes patients feel better
- it may reduce your chance of relapse and improve your overall chance of living a healthy life.

During treatment
It is important to avoid inactivity during treatment. Being active has been shown to improve psychological wellbeing, mood and prevent what is sometimes called ‘chemo brain’.

After treatment
Try to exercise a bit more in an activity you enjoy, such as brisk walking. It is important to build up slowly and incorporate more activity in to your daily life, take the stairs, maybe get off the bus a stop early.

Ian's story

Diagnosed with rectal cancer and secondary liver cancer

“I was diagnosed with a rectal tumour in November 2003 and told it had already spread to my lymph nodes and liver, so it was very serious. I hadn’t shown many symptoms and I assumed it was a haemorrhoid problem but when I saw a new GP I was advised to have the problem checked out. After all the scans and tests, I was told it was cancer and that it was serious. It was devastating at the time as I just didn’t see it coming.

I had people saying to me to be positive but it was frustrating as I wanted to know how I could create a positive attitude, what I could do practically to improve my chances of beating the cancer. How to get a positive mental state is something we could talk about all day but I think it is about small targets, like putting one foot in front of the other, walking ten yards, climbing a flight of stairs.

Throughout my treatment, I just tried to walk whenever I could, even if it was only a little. Even after my operations, I did what I could, with the help of my wife and a nurse. I just felt the need to try and get moving. It felt like I was doing something active rather than being passive and just lying there.

After I left hospital, I carried on walking, with my wife’s help, building up slowly. We would go to the local park and at first I could only walk 100 yards and would have to stop, to sit down, but each day you can do a little more. Gradually I started to feel better. There are some exercises I cannot do due to weakness in my stomach from the operations, but the key is to keep moving.

Eventually, I joined a group called Walking for Health, walking maybe a mile or two, doing that as often as I could. The social side is really good within our group, getting out and meeting people, which is good for you and good for your family too.

It’s good on three levels really: it’s good for your mental attitude, good on the social side and also good for your immune system.”

More stories from people living with cancer >

Incorporate activity in to your everyday life.

A good way to stay motivated is build activity into everyday life. For example, if after a busy day you like to spend time catching up with your friends and family, do so on a brisk walk.

Whatever you decide to do, focus on your positive achievements and remember to use the activity chart in your Move more guide to record and reward your daily achievements.

As well as moving around more, it's also important to reduce time spent sitting. Try standing up for a few minutes at a time to break up long periods sat down. When at work, take phone calls standing up or walk to your colleague's desk rather than sending an email.

Walking is the most popular activity for people who have had a cancer diagnosis. You might find a pedometer useful to track the number of steps that you take each day. You can **download a pedometer** from our 'apps to help you get active page'.

you, or is someone you know, living with cancer?
can help you. Call **0808 808 00 00**
nday - Friday 9am - 8pm) or visit **macmillan.org.uk**

millan Cancer Support, registered charity in England and Wales (261017), Scotland (SC039907) and the Isle of Man (604). A
pany limited by guarantee, registered in England and Wales company number 2400969. Isle of Man company number 4694F.
stered office: 89 Albert Embankment, London SE1 7UQ. /
