# Supplementary material for: The Effect of the Move More Pack on the Physical Activity of Cancer Survivors: Protocol for a Randomized Waiting List Control Trial with Process Evaluation
Source: JMIR Res Protoc. 2017 Nov 9;6(11):e220. doi: 10.2196/resprot.7755 (PMC5701086; doi:10.2196/resprot.7755)
Supplement: Multimedia Appendix 6 [file resprot_v6i11e220_app6.pdf]

TOOLS TO HELP YOU MOVE MORE

Need to talk?
Call us free* >
0808 808 00 00
Monday to Friday, 9am-8pm

Being physically active can help you prevent and manage some of the side effects of treatment, such as fatigue, depression, muscle wasting and risks to your heart health. Choosing to become more active is the first step, but we know it can be hard so we have a range of tools to help you.

Where should I start?

Sign up to our email newsletter >

You will receive six email newsletters over the next 12 months containing expert advice, inspirational quotes, case studies and tips to keep you motivated.

You might also like to:

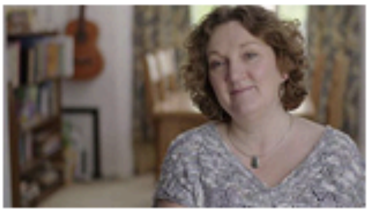

Talk about being active >

Connect with other people on the Online Community and find out what has worked for them.

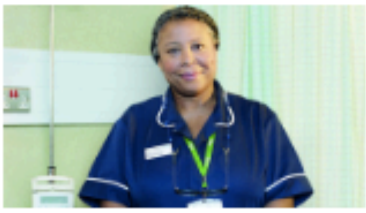

Ask the experts about being active >

Talk to professionals and get practical advice on how to become more active.

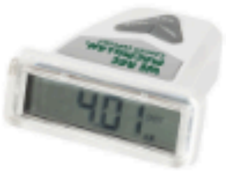

Order a pedometer >

We're with you every step of the way. Keep track of your steps with a Macmillan pedometer.

WE ARE MACMILLAN. CANCER SUPPORT
MOVE MORE

Order free resources >

If you don't have one already, order your free Move More guide. Or download a new activity chart or diary.

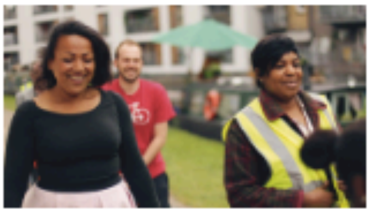

Find activities near you >

Find out about activities in your area, such as walking, gardening and sports, and get involved.

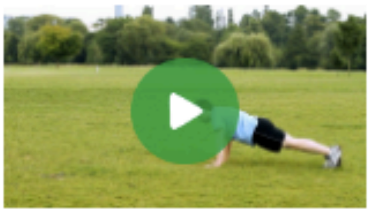

Hear personal stories >

Watch our playlist featuring Ted, Joy and James talking about how physical activity benefited them, and how it can help you.

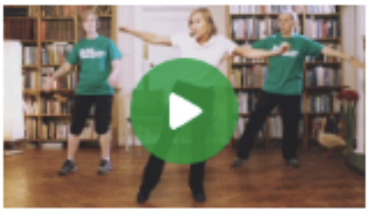

Watch our exercise DVD >

Watch our Move More DVD online, containing activities specifically for people living with or after cancer.

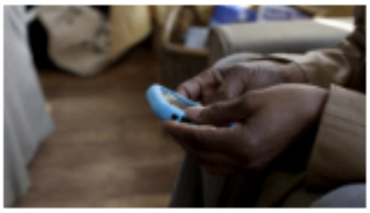

More digital resources >

There are lots of other digital resources and apps available to help you become and stay active before, during and after cancer treatment.
