# Supplementary material for: The Effect of the Move More Pack on the Physical Activity of Cancer Survivors: Protocol for a Randomized Waiting List Control Trial with Process Evaluation
Source: JMIR Res Protoc. 2017 Nov 9;6(11):e220. doi: 10.2196/resprot.7755 (PMC5701086; doi:10.2196/resprot.7755)
Supplement: Multimedia Appendix 7 [file resprot_v6i11e220_app7.pdf]

Multimedia Appendix 7: A breakdown of the components of the revised Move More Pack

Review of the revised Move More Pack against the theoretical constructs of the social cognitive theory (SCT), the theory of planned behavior (TPB), and a review of the behavior change techniques (BCTs) used.

Component of the revised Move More Pack		Section heading(s)	Theoretical constructs	BCT[a,b]
**Printed resource**				
	Guide to becoming active	Why get active?	TPB[c] Behavioral beliefs, normative beliefs<br>SCT[d]- Facilitation	Instructions on how to perform the behavior; credible source
		Case studies	TPB- Behavioral beliefs; normative beliefs; motivation to comply; control beliefs, perceived power<br>SCT- Self-efficacy; observational learning, outcome expectations	Information about health and emotional consequences;<br>social comparison; salience of consequences
		Questions to help you get started	TPB- Evaluations of behavioral outcomes, perceived power<br>SCT- Self-efficacy and outcome expectancy	Comparative imagining of future outcomes; commitment; social support (practical); pros and cons; credible source
		Simple ways to be more active; which activities are right for you? Physical activity in your area	TPB- Control beliefs, perceived power<br>SCT- Self-efficacy; collective-efficacy, facilitation	Instructions on how to perform the behavior; problem-solving; credible source

		Ten top tips	TPB- Evaluations of behavioral outcomes; control beliefs, perceived power<br>SCT- Self-efficacy; collective-efficacy; outcome evaluation; incentive motivation, facilitation	Goal-setting (behavior); self-monitoring of behavior; problem-solving; social support (practical); habit formation; nonspecific reward; credible source
		How to set goals	TPB- Control beliefs and perceived power<br>SCT- Self-efficacy; collective-efficacy; self-regulation, facilitation	Goal-setting (behavior or outcome); problem-solving; credible source
		Track your progress; my activity diary	TPB- Evaluation of behavioral outcomes; control beliefs, perceived power<br>SCT- Self-efficacy; collective-efficacy; self-regulation; incentive motivation, facilitation	Goal-setting (behavior or outcome); commitment; self-monitoring of behavior; monitoring of emotional consequences; credible source; self-reward
		Action plan	TPB- Evaluation of behavioral outcomes; control beliefs, perceived	Action-planning; goal-setting (behavior or outcome); commitment; self-reward; social support (practical); credible source

			power<br>SCT- Self-efficacy; collective-efficacy; self-regulation, facilitation	
	Physical activity and cancer booklet (inserted)	Why be more active? Being active before treatment; being active during treatment; reducing side effects; being active after treatment; advanced cancer and physical activity	TPB- Behavioral beliefs; normative beliefs; motivation to comply, control beliefs<br>SCT- Outcome expectations	Framing or reframing; information about health and emotional consequences; salience of consequences; information about others' approval; credible source
		How much activity is recommended? How much activity is right for you? What types of activity should you do?	TPB- Behavioral beliefs; normative beliefs; motivation to comply, perceived power<br>SCT- Self-efficacy; outcome expectations	Information about health and emotional consequences; salience of consequences; information about others' approval; credible source
		Being safe	TPB- Behavioral beliefs; control beliefs, perceived power<br>SCT- Self-efficacy; outcome expectations	Information about health and emotional consequences; credible source

		Getting started; what activities are near you? Next steps and who can help?	TPB- Normative beliefs; control beliefs, perceived power<br>SCT- Facilitation	Instructions on how to perform the behavior; credible source
	Activity leaflets (inserted)	(1) Activities of daily living; (2) Gardening; (3) Swimming; (4) Walking; (5) Getting back into sport	TPB- Behavioral beliefs; control beliefs, perceived power<br>SCT- Outcome expectations, facilitation	Information about health consequences; credible source
	Pull-out wall chart and fridge magnet (inserted)	Record your daily achievements	TPB- Behavioral beliefs; evaluation of behavioral outcomes; control beliefs, perceived power<br>SCT- Self-efficacy; collective-efficacy; self-regulation, facilitation	Self-monitoring of behavior; salience of consequences; information about others' approval; prompt or cue; self-reward; credible source
	DVD[e] (inserted)	Move More DVD	TPB- Behavioral beliefs; evaluation of behavior outcomes; normative beliefs; control beliefs, perceived power<br>SCT- Self-efficacy; observational learning; outcome expectations; Facilitation,	Instructions on how to perform a behavior; information about health consequences; information about emotional consequences; demonstration of the behavior; social comparison; information about others' approval; graded tasks; credible source

			reciprocal determinism	
**Internet-based tools**				
	Online forum	Talk about being active	TPB- Normative beliefs<br>SCT- Observational learning, facilitation	Social support (practical)
	Online forum	Ask the physio	TPB- Normative beliefs<br>SCT- Observational learning, facilitation	Information about others' approval; social support (practical)
	Digital apps	Apps to help you get active	SCT- Facilitation	Self-monitoring of behavior
	Order a pedometer	Order a pedometer	SCT- Facilitation	Self-monitoring of behavior
	Find activities near you	Activities near you	TPB- Control beliefs<br>SCT- Facilitation	Not applicable
	Video case studies	Hear personal stories	TPB- Behavioral beliefs; normative beliefs; motivation to comply; control beliefs, perceived power<br>SCT- Self-efficacy; observational learning, outcome expectations	Information about health and emotional consequences;<br>social comparison; salience of consequences

	DVD (Web-based)	Watch our exercise DVD	TPB- Behavioral beliefs; evaluation of behavior outcomes; normative beliefs; control beliefs, perceived power SCT- Self-efficacy; observational learning; outcome expectations; Facilitation, reciprocal determinism	Instructions on how to perform a behavior; information about health consequences; information about emotional consequences; demonstration of the behavior; social comparison; information about others' approval; graded tasks; credible source

[a]BCT: behavior change technique.

[b]BCTs selected from the BCT taxonomy version 1.

[c]TPB: theory of planned behavior.

[d]SCT: social cognitive theory.

[e]DVD: digital versatile disc.
