# Supplementary material for: The Effect of the Move More Pack on the Physical Activity of Cancer Survivors: Protocol for a Randomized Waiting List Control Trial with Process Evaluation
Source: JMIR Res Protoc. 2017 Nov 9;6(11):e220. doi: 10.2196/resprot.7755 (PMC5701086; doi:10.2196/resprot.7755)
Supplement: Multimedia Appendix 8 [file resprot_v6i11e220_app8.pdf]

Multimedia Appendix 8: Interview topic guide

Structure

These questions are to be used to lead the discussion. They do not have to be rigidly followed and are for guide purposes only. The interviewer is to use his or her judgement to ensure that information is collected on each topic area.

Introduction:

- Name and role.
- Thank participant for their time.
- Inform the participant that the interview is being recorded and explain how the data will be used.
- Briefly recap the study aims and outcomes.

General questions:

- Please can you tell me a bit about yourself and your cancer journey?
- What are your experiences in being physically active before your cancer diagnosis?
- What are your experiences in being physically active after your diagnosis?
- What motivates you?
- What barriers do you face?
- How have you found the last 12 weeks in regards to your physical activity?
- Tell me about your use of the Move More pack including the online tools?
- Which elements have you found particularly useful?
    - Why? What did you like about it? What encouraged you to use that element? Which particular sections of this element did you find useful and why?
- Which elements did you not find useful?
    - Why? What was it that put you off using this element?

For elements not mentioned during the questioning above, and based on their response to questions in the follow-up questionnaire ask the following:

- Can you tell me why you used [element, including online tools] [often, sometimes, rarely]?

    - How did it help if at all? What did you like / dislike about it? What encouraged / discouraged you to use that element? Which particular sections of this element did you find / not find useful and why?
- Why did you not use the [element, including online tools]?
    - What was it that put you off using this element?

If not already covered:

- What are you views on the e-newsletters? How did they help you, if at all? What stood out? What can you remember of the newsletters?

Views of the Move More pack as a whole:

- What did you think of this information overall? What would you change? Is there anything missing that you would like included?

Conclusion:

- Is there anything that you would like to add or talk about that you think is of relevance to this study?"

Thank participant for their time and inform them of the next steps.
